# Supplementary material for: Novel Biologically Active N-Substituted Benzimidazole Derived Schiff Bases: Design, Synthesis, and Biological Evaluation
Source: Molecules. 2023 Apr 25;28(9):3720. doi: 10.3390/molecules28093720 (PMC10180076; doi:10.3390/molecules28093720)
Supplement: Supplementary file 1 [file molecules-28-03720-s001.zip › molecules-2290879-supplementary.pdf]

Novel biologically active *N*-substituted benzimidazole derived Schiff bases: design, synthesis and biological evaluation

Anja Beč,[1] Maja Cindrić,[1] Leentje Persoons[2], Mihailo Banjanac[3], Vedrana Radovanović[3], Dirk Daelemans[2], and M. Hranjec[1]*

[1] Department of Organic Chemistry, Faculty of Chemical Engineering and Technology, University of Zagreb, Marulićev trg 19, HR-10000 Zagreb, Croatia

[2] KU Leuven, Department of Microbiology, Immunology and Transplantation, Laboratory of Virology and Chemotherapy, Rega Institute, Leuven, Belgium

[3] Pharmacology *in vitro*, Selvita Ltd. Prilaz baruna Filipovića 29, 10000 Zagreb, Croatia

Content

Pages S2-S5: 13C NMR dana and elemntal analysis of prepared compounds

Pages S5-S7: Biological experimental data

Figures S1-S48: NMR spectra of prepared compounds

4.1.2. General method for preparation of compounds 7–8

*N-hexyl-2-nitroaniline **7***

^{13}C NMR (151 MHz, DMSO-d_6): δ/ppm = 145.7, 137.1, 131.3, 126.7, 115.5, 115.0, 42.7, 31.4, 28.7, 26.5, 22.5, 14.4; Anal. Calcd. for $C_{12}H_{18}N_2O_2$: C, 64.84; H, 8.16; N, 12.60; O, 14.39. Found: C, 64.89; H, 8.23; N, 12.51; O, 14.29%.

*3-N-(hexylamino)-4-nitrobenzonitrile **8***

^{13}C NMR (75 MHz, DMSO-d_6): δ/ppm = 146.8, 130.5, 118.2, 96.0, 42.3, 30.8, 27.9, 25.8, 21.9; Anal. Calcd. for $C_{13}H_{17}N_3O_2$: C, 63.14; H, 6.93; N, 16.99; O, 12.94. Found: C, 63.20; H, 6.88; N, 16.91; O, 12.89%.

4.1.3. General method for preparation of compounds 15–16

*N^1-hexylbenzene-1,2-diamine **15***

^{13}C NMR (151 MHz, DMSO-d_6): δ/ppm = 136.1, 135.0, 117.5, 116.5, 114.0, 109.6, 43.4, 31.2, 28.8, 26.5, 22.1, 13.9; Anal. Calcd. for $C_{12}H_{20}N_2$: C, 74.95; H, 10.48; N, 14.57. Found: C, 74.88; H, 10.54; N, 14.66%.

*3-amino-4-N-(hexylamino)benzonitrile **16***

^{13}C NMR (75 MHz, DMSO-d_6): δ/ppm = 140.5, 135.5, 123.4, 121.6, 115.2, 108.8, 96.7, 43.2, 31.6, 28.8, 26.8, 22.6, 14.4; Anal. Calcd. for $C_{13}H_{19}N_3$: C, 71.85; H, 8.81; N, 19.34. Found: C, 71.94; H, 8.87; N, 19.41%.

4.1.4. General method for preparation of compounds 23 and 24

*2-amino-1-hexylbenzimidazole **23***

^{13}C NMR (151 MHz, DMSO-d_6): δ/ppm = 151.6, 131.9, 122.9, 121.9, 112.9, 109.9, 42.4, 31.3, 28.2, 26.0, 22.5, 14.3; Anal. Calcd. for $C_{13}H_{19}N_3$: C, 71.85; H, 8.81; N, 19.34. Found: C, 71.87; H, 8.79; N, 19.29%.

*2-amino-6-cyano-1-hexylbenzimidazole **24***

^{13}C NMR (151 MHz, DMSO-d_6): δ/ppm = 157.1, 143.2, 138.3, 121.2, 102.5, 42.1, 31.4, 28.7, 26.1, 22.5; Anal. Calcd. for $C_{14}H_{18}N_4$: C, 69.39; H, 7.49; N, 23.12. Found: C, 69.45; H, 7.40; N, 23.07%.

4.1.5. General method for preparation of Schiff bases 28–45

(E)-4-(((1-isobutyl-1H-benzo[d]imidazol-2-yl)imino)methyl)-N,N-dimethylaniline ***28***

^{13}C NMR (151 MHz, DMSO-*d*$_6$): δ/ppm = 150.4, 131.2 (2C), 129.1 (2C), 124.0, 123.4, 112.0 (2C), 111.2 (3C), 49.1, 27.7, 19.7 (2C); Anal. Calcd. for $C_{20}H_{24}N_4$: C, 74.97; H, 7.55; N, 17.48. Found: C, 74.95; H, 7.59; N, 17.41%.

(E)-2-((4-(dimethylamino)benzylidene)amino)-1-isobutyl-1H-benzo[d]imidazole-6-carbonitrile ***29***

^{13}C NMR (100 MHz, DMSO-*d*$_6$) (δ/ppm): 166.2, 159.0, 154.3, 141.6, 138.7, 132.6, 125.2, 122.7, 120.6, 112.1, 104.1, 49.8 , 29.3, 20.3; Anal. Calcd. for $C_{21}H_{23}N_5$: C, 73.02; H, 6.71; N, 20.27. Found: C, 73.10; H, 6.68; N, 20.32%.

(E)-5-(diethylamino)-2-(((1-isobutyl-1H-benzo[d]imidazol-2-yl)imino)methyl)phenol ***30***

^{13}C NMR (151 MHz, DMSO-d$_6$): δ/ppm = 163.5, 153.8, 152.9, 135.0, 121.8, 121.3, 117.9, 111.2, 110.0, 108.5, 105.0, 104.4, 96.6, 95.9, 49.4, 44.1 (2C), 28.9, 19.9 (2C), 12.5 (2C); Anal. Calcd. for $C_{22}H_{28}N_4O$: C, 72.50; H, 7.74; N, 15.37; O, 4.39. Found: C, 72.48; H, 7.70; N, 15.32; O, 4.44%.

(E)-2-((4-(diethylamino)-2-hydroxybenzylidene)amino)-1-isobutyl-1H-benzo[d]imidazole-6-carbonitrile ***31***

^{13}C NMR (151 MHz, DMSO-*d*$_6$): δ/ppm = 164.3, 153.9 (2C), 141.6, 138.7, 125.3, 122.6, 120.6, 111.8 (2C), 109.1, 105.9 (2C), 104.1, 97.0 (2C), 50.0, 44.7, 29.4, 20.3, 13.0; Anal. Calcd. for $C_{23}H_{27}N_5O$: C, 70.92; H, 6.99; N, 17.98; O, 4.11. Found: C, 70.97; H, 6.91; N, 17.93; O, 4.08%.

(E)-1-isobutyl-2-((4-nitrobenzylidene)amino)-1H-benzo[d]imidazole-6-carbonitrile ***32***

^{13}C NMR (151 MHz, DMSO-*d*$_6$) (δ/ppm): 165.2, 156.2, 149.8, 140.6, 140.2, 138.2, 130.9 (2C), 125.7, 124.2 (2C), 123.9, 119.8, 112.4, 104.6, 49.6, 29.0, 19.8 (2C); Anal. Calcd. for $C_{19}H_{17}N_5O_2$: C, 65.69; H, 4.93; N, 20.16; O, 9.21. Found: C, 65.72; H, 4.87; N, 20.09; O, 9.34%.

(E)-N,N-dimethyl-4-(((1-methyl-1H-benzo[d]imidazol-2-yl)imino)methyl)aniline ***33***

^{13}C NMR (151 MHz, DMSO-d$_6$): δ/ppm = 190.4, 154.7, 132.0, 125.0, 123.8, 123.1, 112.2, 112.1, 111.6, 111.0, 110.4, 19.8, 9.1 (2C); Anal. Calcd. for $C_{17}H_{18}N_4$: C, 71.27; H, 5.65; N, 23.09. Found: C, 71.30; H, 5.61; N, 23.16%.

(E)-2-((4-(dimethylamino)benzylidene)amino)-1-methyl-1H-benzo[d]imidazole-6-carbonitrile ***34***

^{13}C NMR (100 MHz, DMSO-d_6) (δ/ppm): 166.4, 158.9, 154.3, 141.7, 139.0, 132.8, 125.2, 122.9, 122.8, 122.6, 120.7, 118.2, 112.0, 111.6, 108.8, 104.1, 29.5 (2C), 29.1; Anal. Calcd. for $C_{18}H_{17}N_5$: C, 71.27; H, 5.65; N, 23.09. Found: C, 71.30; H, 5.61; N, 23.16%.

(E)-5-(diethylamino)-2-(((1-methyl-1H-benzo[d]imidazol-2-yl)imino)methyl)phenol ***35***

^{13}C NMR (151 MHz, DMSO-d_6): δ/ppm = 164.5, 163.9, 153.4, 141.9 (2C), 135.8, 122.3, 121.7, 118.3, 110.2, 109.1, 105.5, 97.0, 44.6 (2C), 29.1, 13.0 (2C); Anal. Calcd. for $C_{19}H_{22}N_4O$: C, 70.78; H, 6.88; N, 17.38; O, 4.96. Found: C, 70.71; H, 6.79; N, 17.44; O, 5.03%.

(E)-2-((4-(diethylamino)-2-hydroxybenzylidene)amino)-1-methyl-1H-benzo[d]imidazole-6-carbonitrile ***36***

^{13}C NMR (100 MHz, DMSO-d_6) (δ/ppm): 165.3, 164.2, 157.6, 153.9, 141.7, 138.9, 125.2, 122.4, 120.7, 111.5, 109.2, 105.9, 104.1, 96.9, 44.7 (2C), 29.5, 13.0 (2C); Anal. Calcd. for $C_{20}H_{21}N_5O$: C, 69.14; H, 6.09; N, 20.16; O, 4.61. Found: C, 69.19; H, 5.98; N, 20.13; O, 4.71%.

(E)-1-methyl-2-((4-nitrobenzylidene)amino)-1H-benzo[d]imidazole-6-carbonitrile ***37***

^{13}C NMR (151 MHz, DMSO-d_6): δ/ppm = 165.9, 156.7, 150.4, 141.2, 140.7, 139.1, 131.5 (2C), 126.2, 124.7 (2C), 124.3, 120.3, 112.6, 105.1, 29.9; Anal. Calcd. for $C_{16}H_{11}N_5O_2$: C, 62.95; H, 3.63; N, 22.94; O, 10.48. Found: C, 62.91; H, 3.69; N, 22.88; O, 10.41%.

(E)-N,N-dimethyl-4-(((1-phenyl-1H-benzo[d]imidazol-2-yl)imino)methyl)aniline ***38***

^{13}C NMR (151 MHz, DMSO-d_6): δ/ppm = 165.5, 156.2, 153.9, 142.1, 135.9, 135.6, 132.3, 129.7 (2C), 128.2, 127.4 (2C), 123.1, 122.9, 122.6, 118.9, 112.0, 110.4; Anal. Calcd. for $C_{22}H_{20}N_4$: C, 77.62; H, 5.92; N, 16.46. Found: C, 77.68; H, 5.98; N, 16.39%.

(E)-2-((4-(dimethylamino)benzylidene)amino)-1-phenyl-1H-benzo[d]imidazole-6-carbonitrile ***39***

^{13}C NMR (151 MHz, DMSO-d_6): δ/ppm = 167.1, 158.6, 154.4, 141.9, 138.6, 135.0, 129.8 (2C), 128.8, 127.5 (2C), 126.1, 123.2, 122.6, 120.4, 112.0, 111.7, 105.0; Anal. Calcd. for $C_{23}H_{19}N_5$: C, 75.59; H, 5.24; N, 19.16. Found: C, 75.65; H, 5.30; N, 19.09%.

(E)-5-(diethylamino)-2-(((1-phenyl-1H-benzo[d]imidazol-2-yl)imino)methyl)phenol ***40***

^{13}C NMR (75 MHz, DMSO-d_6) (δ/ppm): 164.4, 163.5, 153.0, 141.6, 135.6, 135.1, 135.0, 129.7 (2C), 128.2, 126.9 (2C), 122.6, 122.1, 118.3, 109.7, 108.5, 105.0, 96.5, 44.1, 12.5; Anal. Calcd. for $C_{24}H_{24}N_4O$: C, 74.97; H, 6.29; N, 14.57; O, 4.16. Found: C, 74.89; H, 6.19; N, 14.51; O, 4.20%.

(E)-2-((4-(diethylamino)-2-hydroxybenzylidene)amino)-1-phenyl-1H-benzo[d]imidazole-6-carbonitrile ***41***

^{13}C NMR (100 MHz, DMSO-*d*$_6$) (δ/ppm): 165.8, 164.4, 156.8, 154.0, 142.0, 138.6, 136.4, 134.7, 130.3, 129.3, 127.8, 126.2, 122.9, 120.4, 111.5, 109.1, 105.9, 105.0, 96.9, 44.7 (2C), 13.0 (2C); Anal. Calcd. for $C_{25}H_{23}N_5O$: C, 73.33; H, 5.66; N, 17.10; O, 3.91. Found: C, 73.36; H, 5.69; N, 17.15; O, 4.02%.

(E)-4-(((1-hexyl-1H-benzo[d]imidazol-2-yl)imino)methyl)-N,N-dimethylaniline ***42***

42a:^{13}C NMR (151 MHz, DMSO-*d*$_6$) (δ/ppm): 165.8, 153.8, 137.8, 124.7, 122.4 (2C), 122.4, 122.2, 120.1, 117.7, 111.5 (2C), 111.1 (2C), 108.4, 103.6, 41.9, 30.5, 28.9, 25.5, 21.9, 13.8; **42b**: ^{13}C NMR (151 MHz, DMSO-*d*$_6$) (δ/ppm): 156.6, 142.8, 141.2 (2C), 132.1, 123.0, 128.1, 122.4 (2C), 120.7, 117.7 (2C), 111.1, 111.0, 102.0, 41.6, 30.9, 28.2, 25.6, 22.0, 13.8; Anal. Calcd. for $C_{22}H_{28}N_4$: C, 75.82; H, 8.10; N, 16.08. Found: C, 75.78; H, 8.16; N, 16.14%.

(E)-2-((4-(dimethylamino)benzylidene)amino)-1-hexyl-1H-benzo[d]imidazole-6-carbonitrile ***43***

^{13}C NMR (151 MHz, DMSO-*d*$_6$) (δ/ppm): 165.77, 158.29, 156.63, 153.81, 142.75, 141.22, 137.80, 137.78, 132.15, 129.99, 128.12, 124.68, 122.38, 122.35, 122.20, 120.65, 120.12, 117.68, 111.64, 111.54, 111.14, 111.03, 108.41, 103.57, 101.96, 41.96, 41.60, 30.85, 30.50, 28.90, 28.23, 25.57, 25.50, 21.96, 21.90, 13.80, 13.75; Anal. Calcd. for $C_{23}H_{27}N_5$: C, 73.96; H, 7.29; N, 18.75. Found: C, 74.01; H, 7.24; N, 18.70%.

(E)-5-(diethylamino)-2-(((1-hexyl-1H-benzo[d]imidazol-2-yl)imino)methyl)phenol ***44***

^{13}C NMR (100 MHz, DMSO-*d*$_6$) (δ/ppm): 164.0, 163.9, 154.6, 154.3, 153.4, 141.9, 135.1, 122.2, 121.8, 118.4, 111.7, 110.2, 109.0, 105.5, 104.9, 97.0, 96.4, 44.6, 44.6, 42.6, 31.2, 29.6, 26.4, 22.5, 14.2, 13.0, 12.9; Anal. Calcd. for $C_{24}H_{32}N_4O$: C, 73.43; H, 8.22; N, 14.27; O, 4.08. Found: C, 73.37; H, 8.09; N, 14.15; O, 4.02%.

(E)-2-((4-(diethylamino)-2-hydroxybenzylidene)amino)-1-hexyl-1H-benzo[d]imidazole-6-carbonitrile ***45***

^{13}C NMR (151 MHz, DMSO-*d*$_6$) (δ/ppm): 164.3, 153.9, 141.7, 138.3, 125.2, 122.6, 120.6, 111.5, 109.1, 105.9 (2C), 104.1, 96.9 (2C), 44.7 (2C), 42.9, 31.1, 29.5, 26.3, 22.4, 14.2, 13.0; Anal. Calcd. for $C_{25}H_{31}N_5O$: C, 71.91; H, 7.48; N, 16.77; O, 3.83. Found: C, 71.94; H, 7.42; N, 16.71; O, 3.88%.

4.2. Biology

4.2.1. Antiviral activity

HEL 299 (ATCC CCL-137; human lung fibroblast), Huh-7 (CLS – 300156; human hepatoblastoma), and MDCK (Madin-Darby canine kidney cells; a kind gift from M. Matrosovich, Marburg, Germany) were maintained in Dulbecco's Modified Eagle Medium (DMEM, Gibco Life Technologies) supplemented with 8% heat-inactivated fetal bovine serum (HyClone, GE Healthcare Life Sciences), 0.075% sodium bicarbonate (Gibco Life Technologies) and 1mM sodium pyruvate (Gibco Life Technologies), and maintained at 37°C under 5% CO_2. Antiviral assays towards herpes simplex virus-1 (HSV-1 KOS), human coronavirus (HCoV-229E and -OC43) and respiratory syncytial virus A in HEL 299 cell cultures, sindbis virus, yellow fever virus, Zika virus and human coronavirus (HCoV-NL63) in Huh-7 cell cultures and influenza A/H1N1 (A/Ned/378/05), influenza A/H3N2 (A/HK/7/87), influenza B (B/Ned/537/05) in MDCK cell cultures were performed. On the day of the infection, growth medium was aspirated and replaced by serial dilutions of the test compounds. The virus was then added to each well, diluted to obtain a viral input of 100 $CCID_{50}$ ($CCID_{50}$ being the virus dose that is able to infect 50% of the cell cultures). Mock-treated cultures receiving solely the test compounds were included, to determine the cytotoxicity. After 3 to 7 days of incubation, the virus-induced cytopathogenic effect was measured colorimetrically by the formazan-based MTS cell viability assay (CellTiter 96 AQueous One Solution Cell Proliferation Assay from Promega, Madison, WI), and the antiviral activity was expressed as the 50% effective concentration (EC_{50}). In parallel, the 50% cytotoxic concentration (CC_{50}) was derived from the mock-infected cells. The activities were compared with the activities of reference antiviral drugs: remdesivir, ribavirin, zanamivir, rimantadine and brivudine (BVDU).

4.2.2. Antibacterial activity

4.2.2.1. Materials

In addition to the synthesized compounds, standard antibiotics ampicillin, ceftazidime, ciprofloxacin and meropenem from USP were tested. Selected bacterial strains were gram negative *E. coli*, *K. pneumoniae*, *A. baumannii* and *P. aeruginosa* and gram positive *S. aureus*, S. *pneumoniae* and *E. faecalis*. Synthesized compounds were prepared as 10 mM DMSO solutions and tested in a final concentration range of 100 - 0.2 µM.[i]

Standard antibiotics were prepared as 5 mg/mL DMSO solutions and tested in a final concentration range of 64 – 0.125 µg/mL.

4.2.2.2. Methods

Broth microdilution testing was performed according to CLSI (Clinical Laboratory Standards Institute) guidelines. MIC (minimal inhibitory concentration) value was defined as the last tested concentration of compound at which there is no visible growth of bacteria. Inoculums for each microorganism were prepared using the direct colony suspension method where broth solutions that achieved turbidity equivalent to 0.5 McFarland standard were additionally diluted 100x with Ca adjusted MH media (Becton Dickinson). All test plates were incubated for 16 - 24h at 37 °C. MIC values for reference antibiotics against quality control strains were used for confirming the validity of the screen according to the Clinical and Laboratory Standards Institute (CLSI) guidelines. Methods for dilution antimicrobial susceptibility tests for bacteria that grow aerobically M07, 11th edition, 2018. and Clinical and Laboratory Standards Institute (CLSI) guidelines. Performance standards for antimicrobial susceptibility testing M100, 28th edition, 2018.

4.2.3 Cell culture and reference compounds

Human cancer cells used in this manuscript, namely Capan-1, HCT-116, NCI-H460, LN-229, HL-60, K-562 and Z-138 were acquired from the American Type Culture Collection (ATCC, Manassas, VA, USA), while the DND-41 cell line was purchased from the Deutsche Sammlung von Mikroorganismen und Zellkulturen (DSMZ Leibniz-Institut, Germany). Culture media were purchased from Gibco Life Technologies, USA, and supplemented with 10% fetal bovine serum (HyClone, GE Healthcare Life Sciences, USA). Vincristine and *docetaxel*, which were used as reference inhibitors, were purchased from Selleckchem (Munich, Germany). Stock solutions were prepared in DMSO.

4.2.4 Proliferation assays

Adherent cell lines LN-229, HCT-116, NCI-H460 and Capan-1 cells were seeded at a density between 500 and 1500 cells per well, in 384-well tissue culture plates (Greiner). After overnight incubation, cells were treated with seven different concentrations of the test compounds, ranging from 100 to 0.006µM. Suspension cell lines HL-60, K-562, Z-138 and DND-41 were seeded at densities ranging from 2500 to 5500 cells per well in 384-well culture plates containing the test compounds at the same concentration points.

Cells were incubated for 72 hours with compounds and were then analysed using the CellTiter 96® AQueous One Solution Cell Proliferation Assay (MTS) reagent (Promega) according to the manufacturer's instructions. Absorbance of the samples was measured at 490 nm using a SpectraMax Plus 384 (Molecular Devices), and OD values were used to calculate the 50% inhibitory concentration (IC_{50}). Compounds were tested in two independent experiments.

Figure S1. ^{1}H NMR spectrum (DMSO-d_6, 400 MHz) of ***N-hexyl-2-nitroaniline 7***

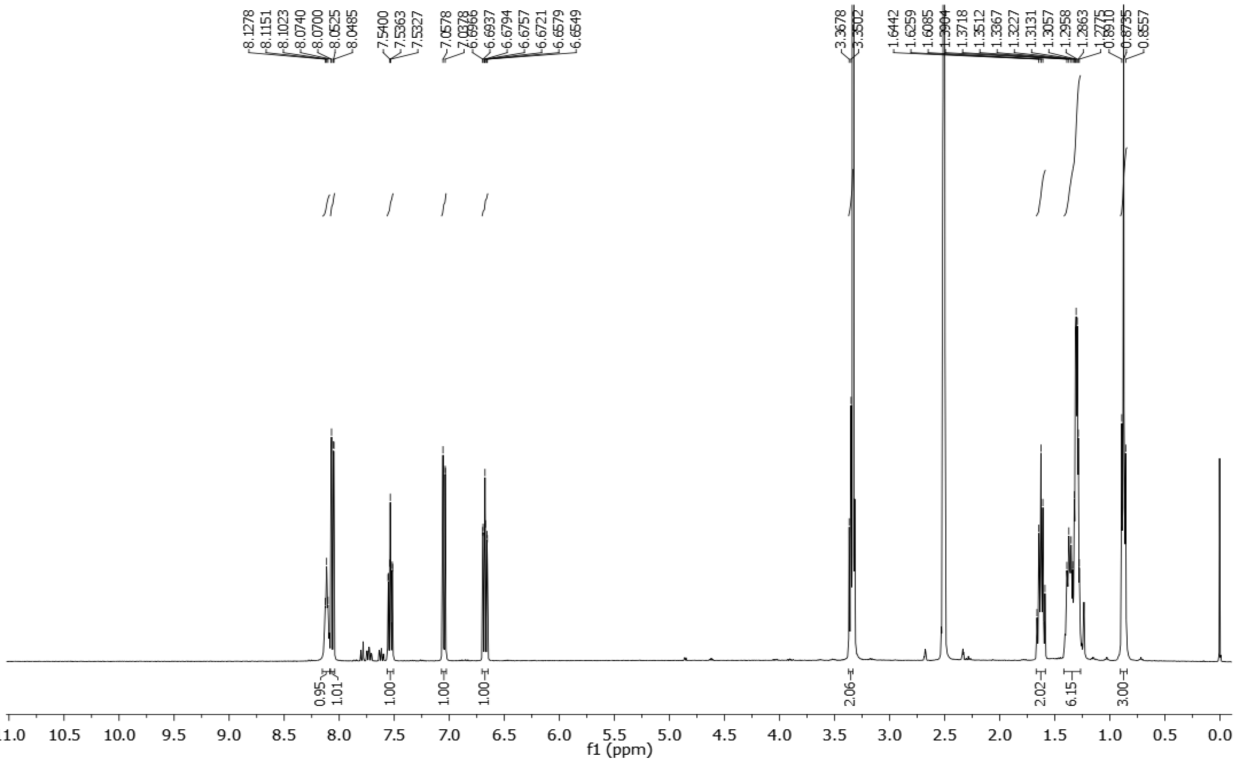

Figure S2. ^{13}C NMR spectrum (DMSO-d_6, 151 MHz) of ***N-hexyl-2-nitroaniline 7***

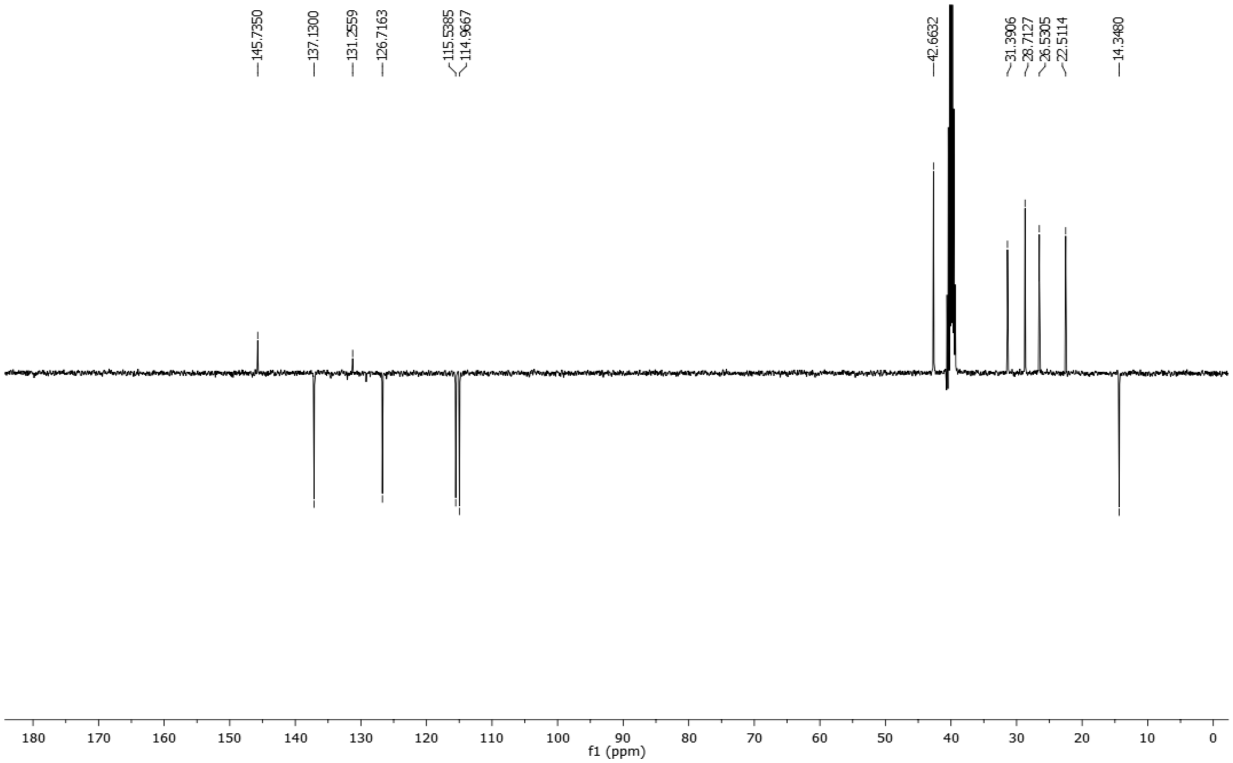

Figure S3. ^{1}H NMR spectrum (DMSO-d_6, 400 MHz) of ***3-N-(hexylamino)-4-nitrobenzonitrile 8***

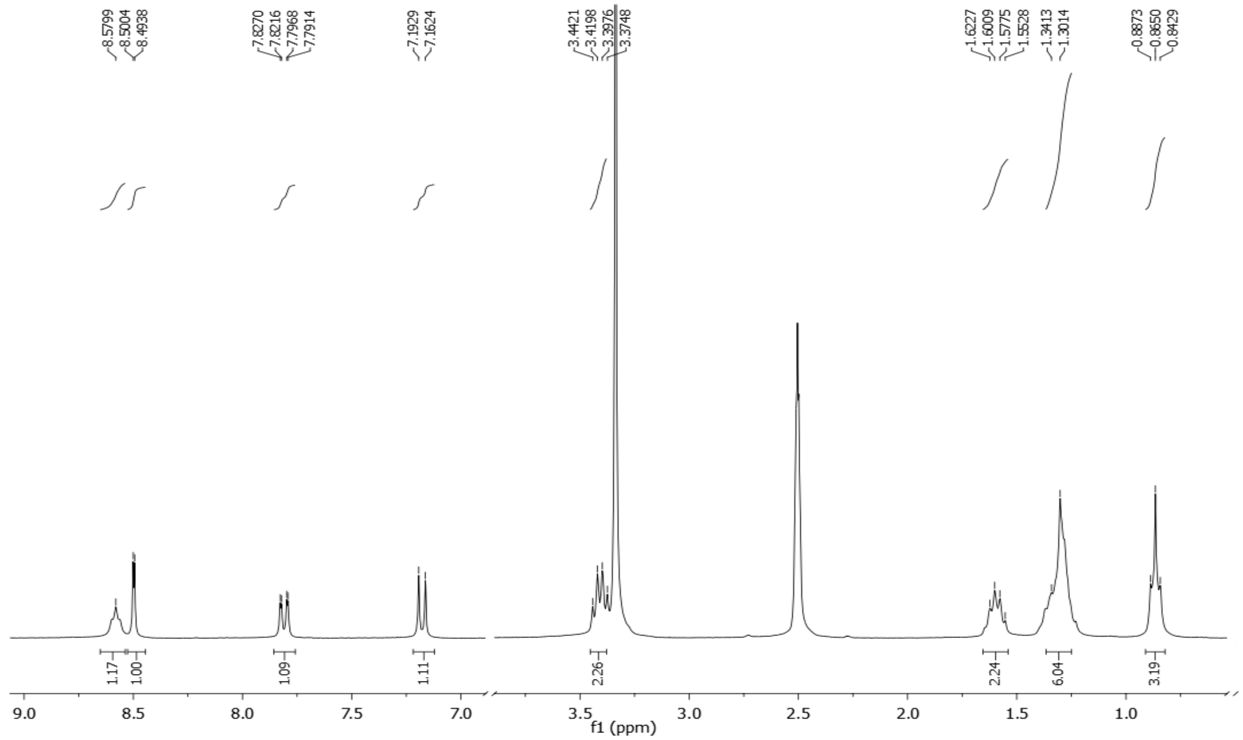

Figure S4. ^{13}C NMR spectrum (DMSO-d_6, 75 MHz) of ***3-N-(hexylamino)-4-nitrobenzonitrile 8***

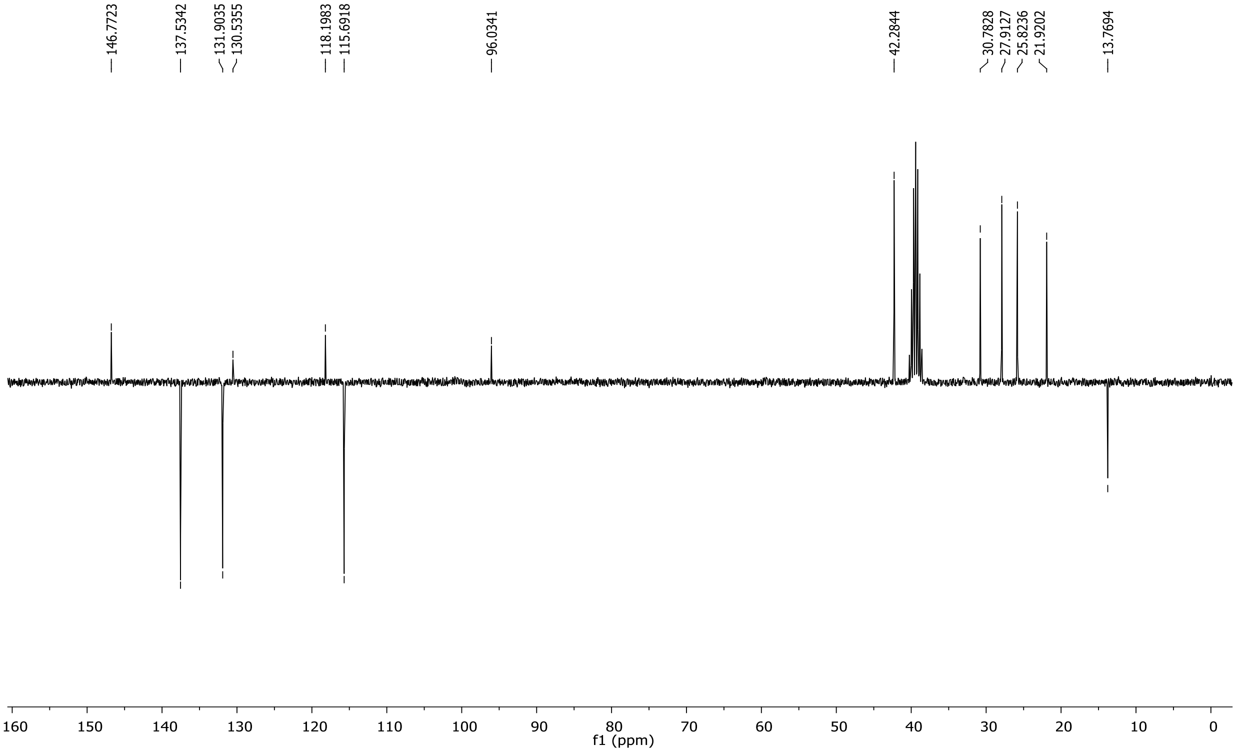

Figure S5. ^{1}H NMR spectrum (DMSO-d_6, 600 MHz) of ***N^{1}-hexylbenzene-1,2-diamine 10***

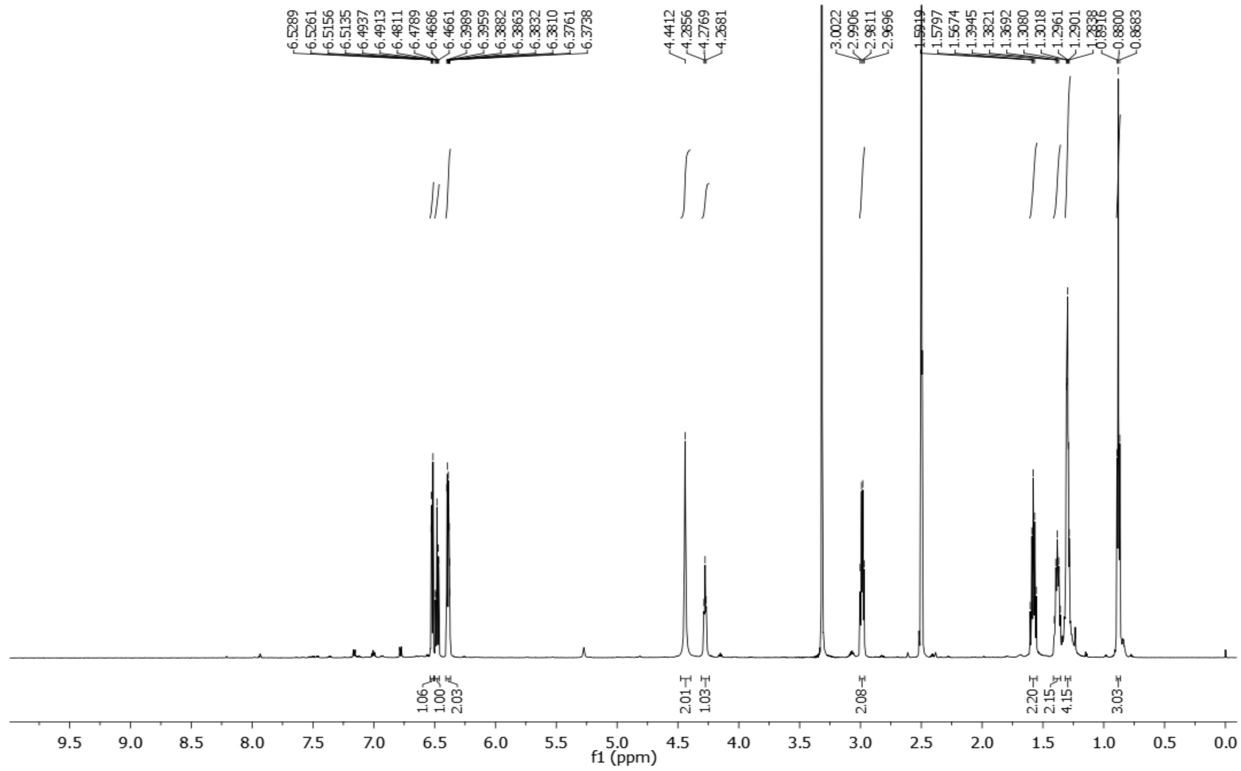

Figure S6. ^{13}C NMR spectrum (DMSO-d_6, 151 MHz) of ***N*[1]-hexylbenzene-1,2-diamine 10**

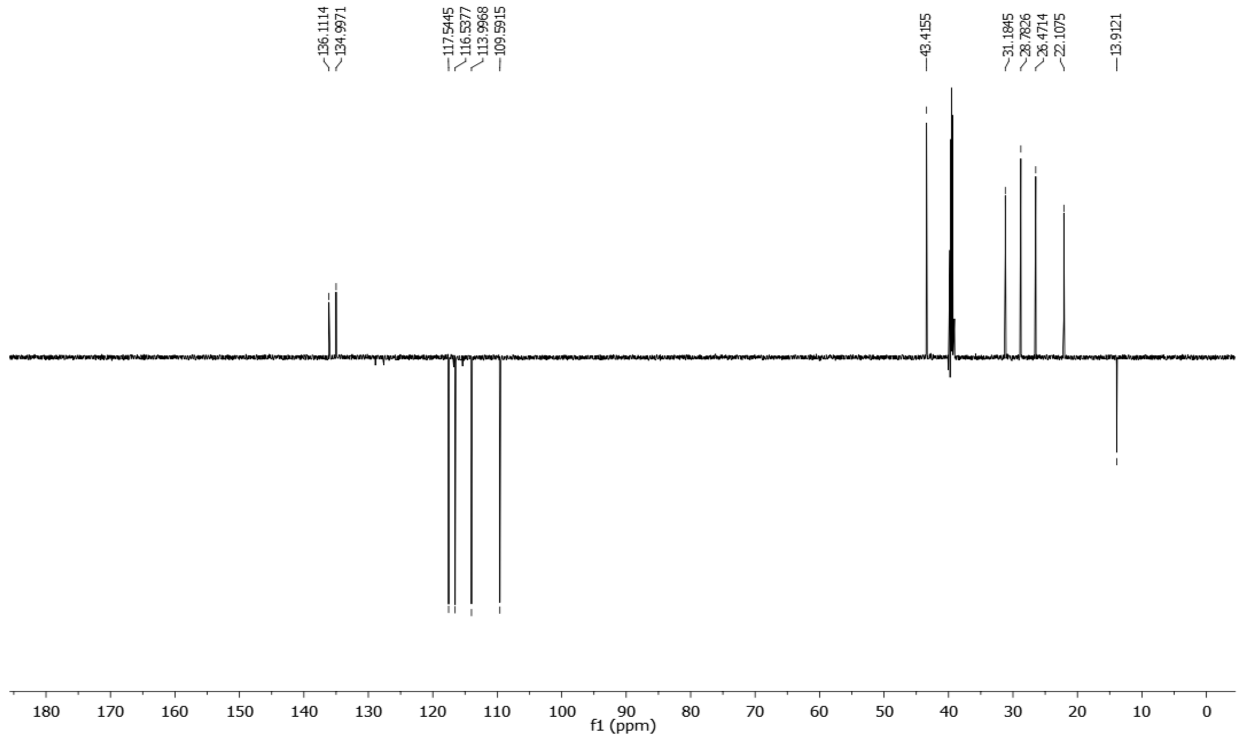

Figure S7. ^{1}H NMR spectrum (DMSO-d_6, 600 MHz) of ***3-amino-4-N-(hexylamino)benzonitrile 16***

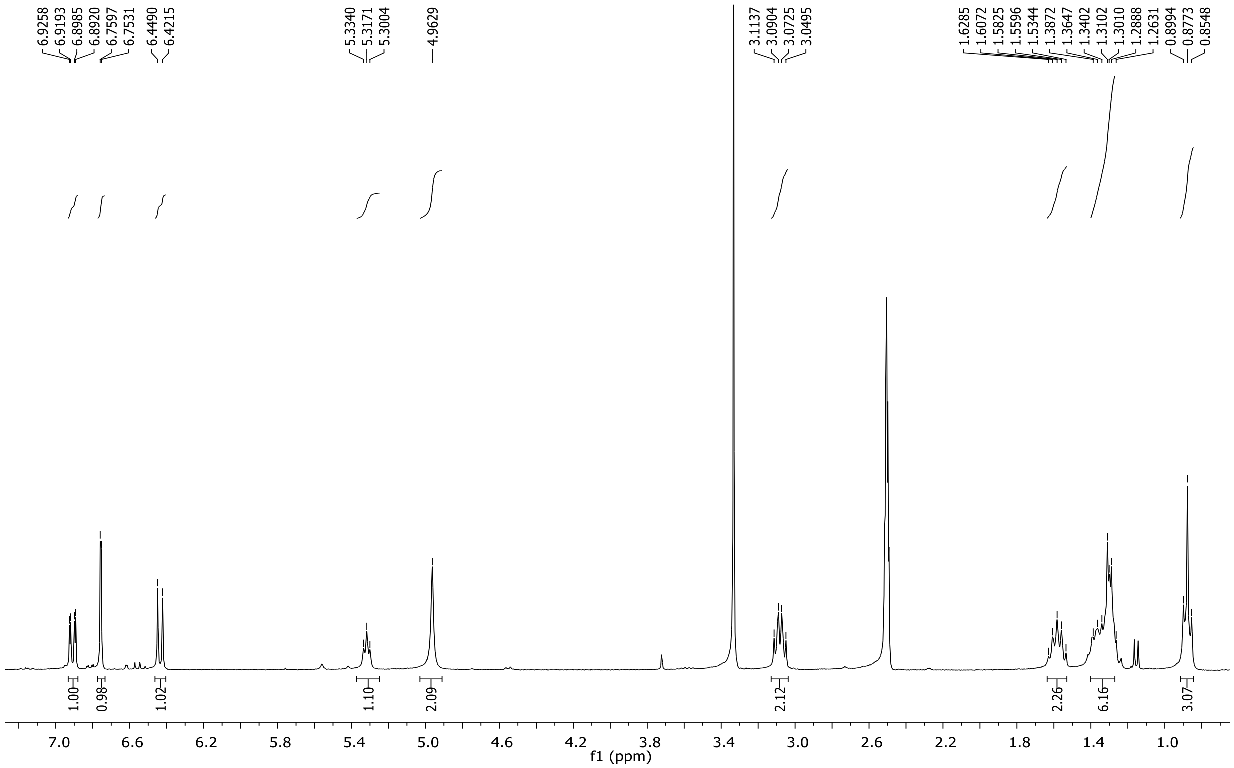

Figure S8. ^{13}C NMR spectrum (DMSO-d_6, 75 MHz) of ***3-amino-4-N-(hexylamino)benzonitrile 16***

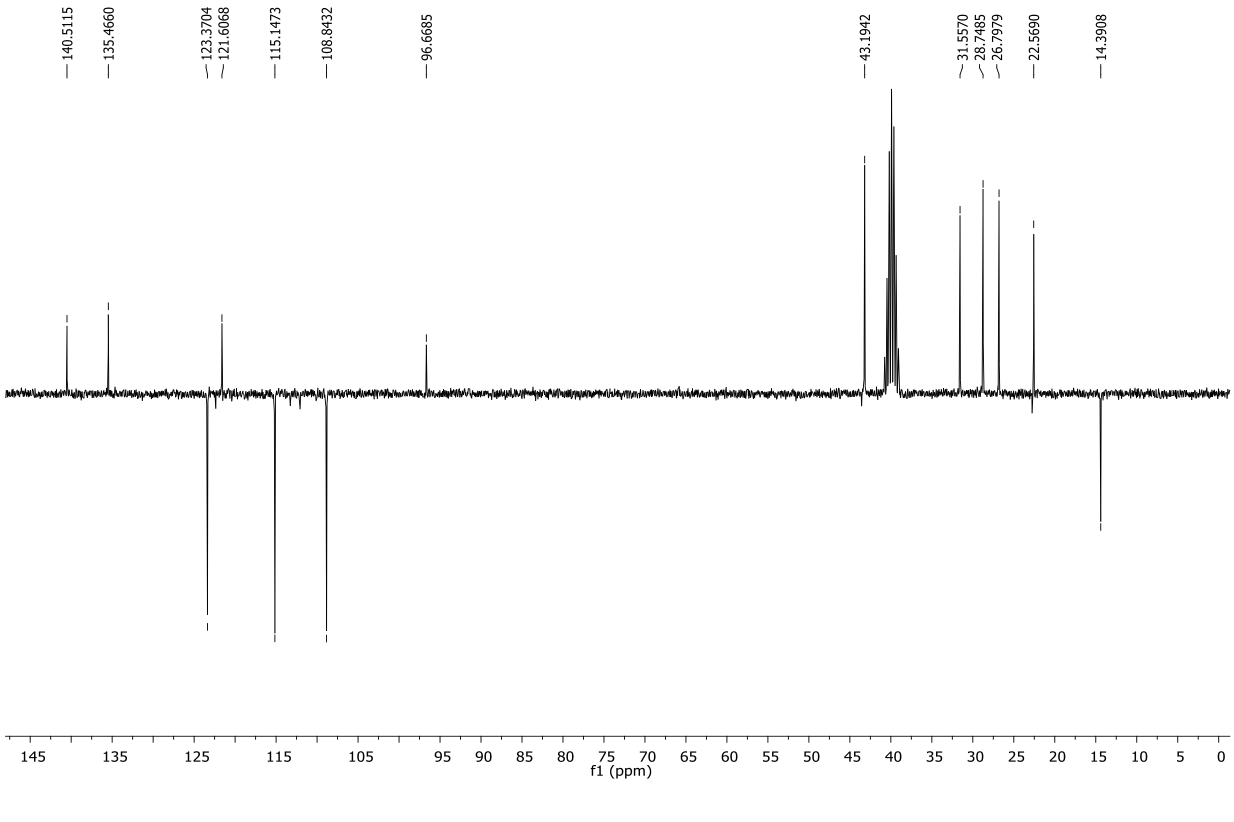

Figure S9. ^{1}H NMR spectrum (DMSO-d_6, 600 MHz) of ***2-amino-1-hexylbenzimidazole 23***

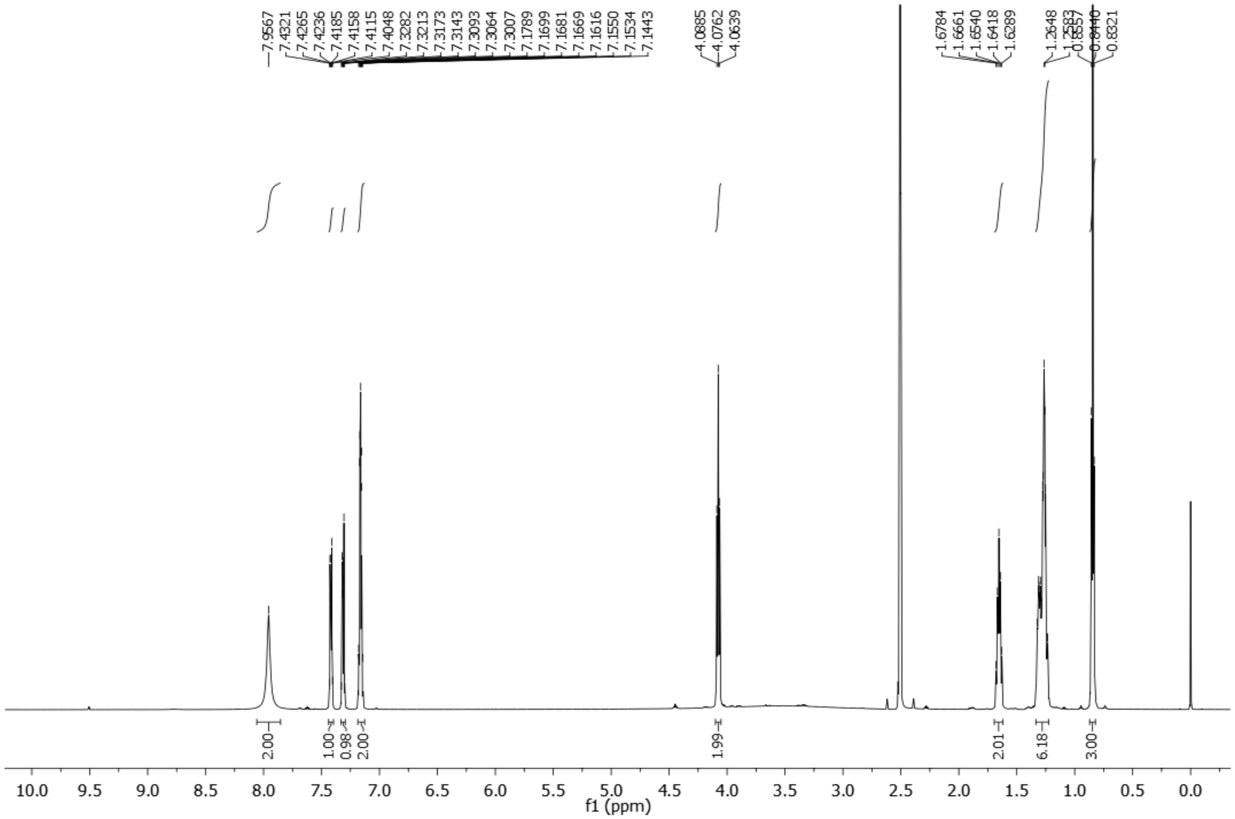

Figure S10. ^{13}C NMR spectrum (DMSO-d_6, 151 MHz) of ***2-amino-1-hexylbenzimidazole 23***

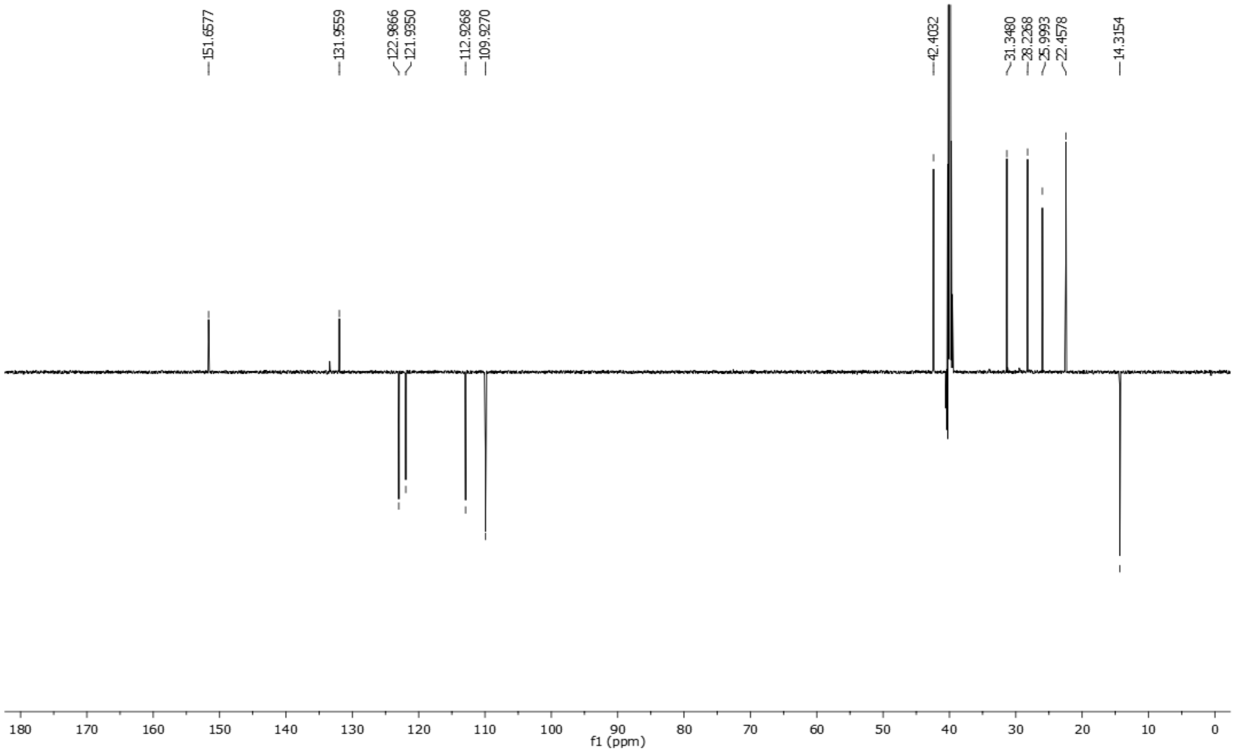

Figure S11. ^{1}H NMR spectrum (DMSO-d_6, 600 MHz) of ***2-amino-6-cyano-1-hexylbenzimidazole 24***

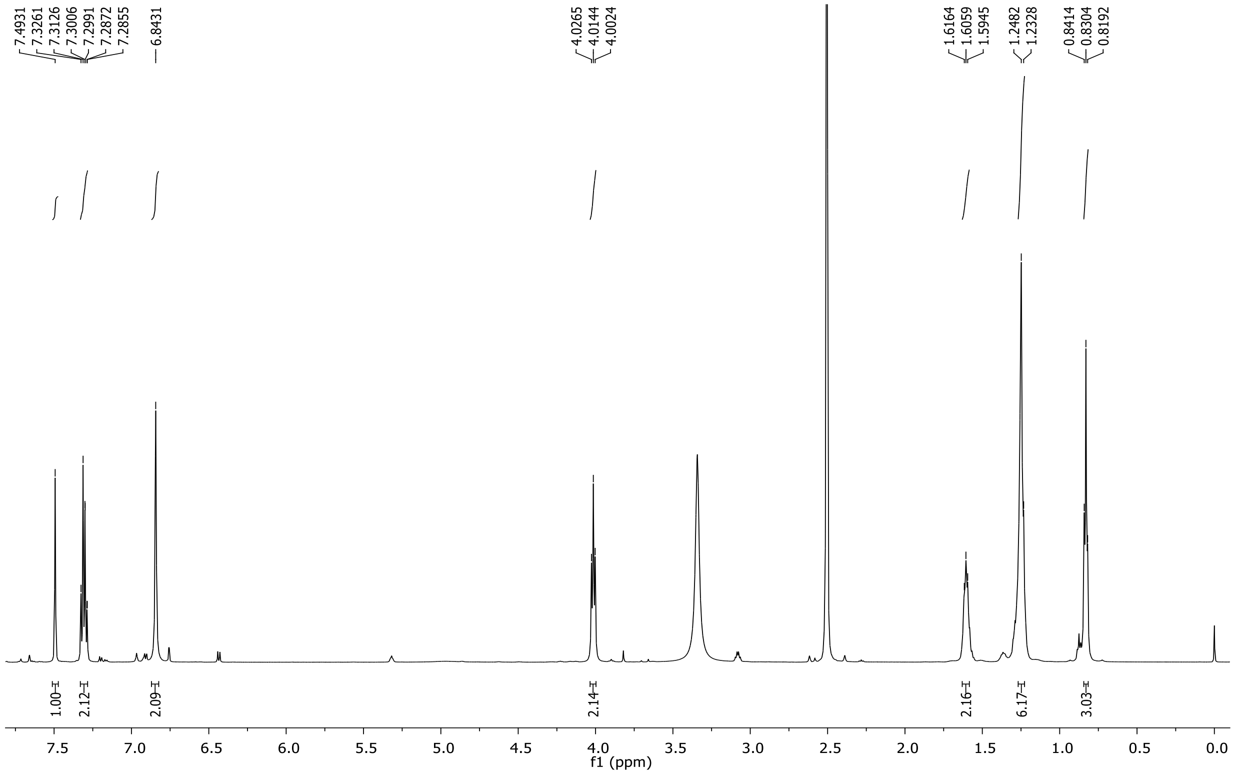

Figure S12. ^{13}C NMR spectrum (DMSO-d_6, 151 MHz) of ***2-amino-6-cyano-1-hexylbenzimidazole 24***

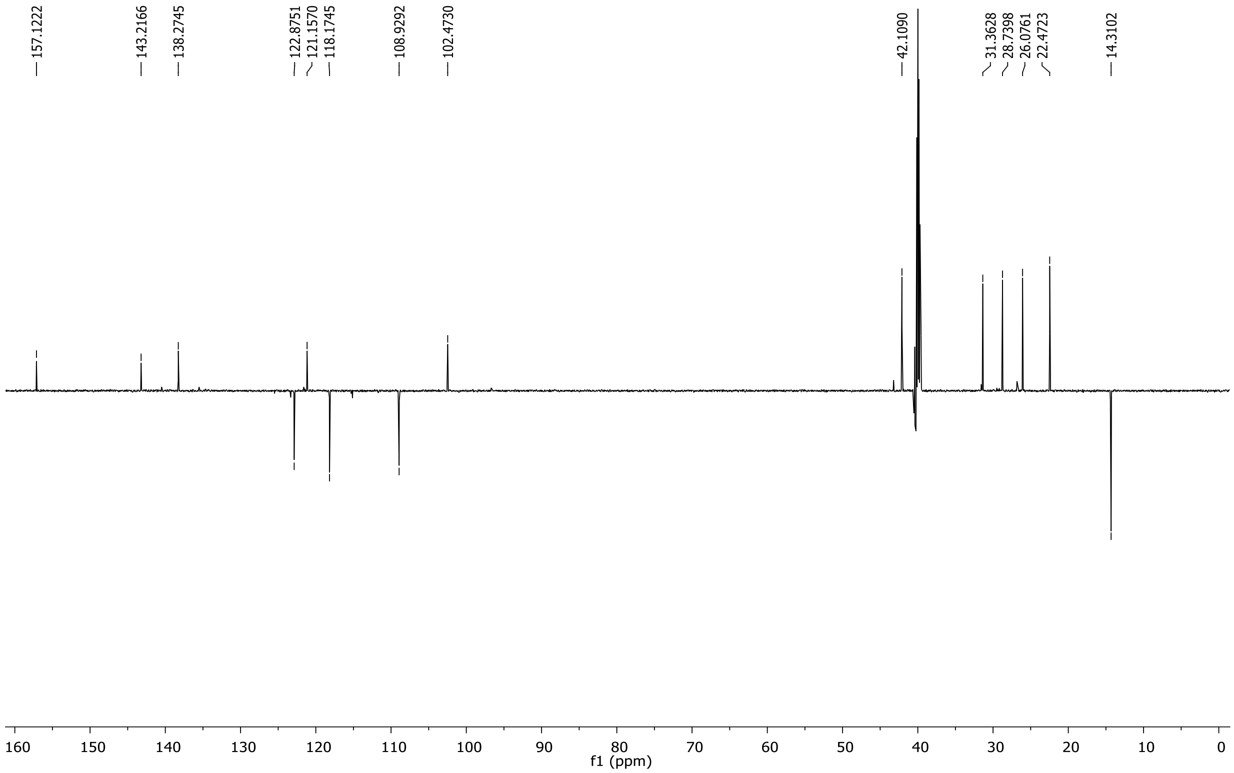

Figure S13. ^{1}H NMR spectrum (DMSO-d_6, 600 MHz) of ***(E)-4-(((1-isobutyl-1H-benzo[d]imidazol-2-yl)imino)methyl)-N,N-dimethylaniline 28***

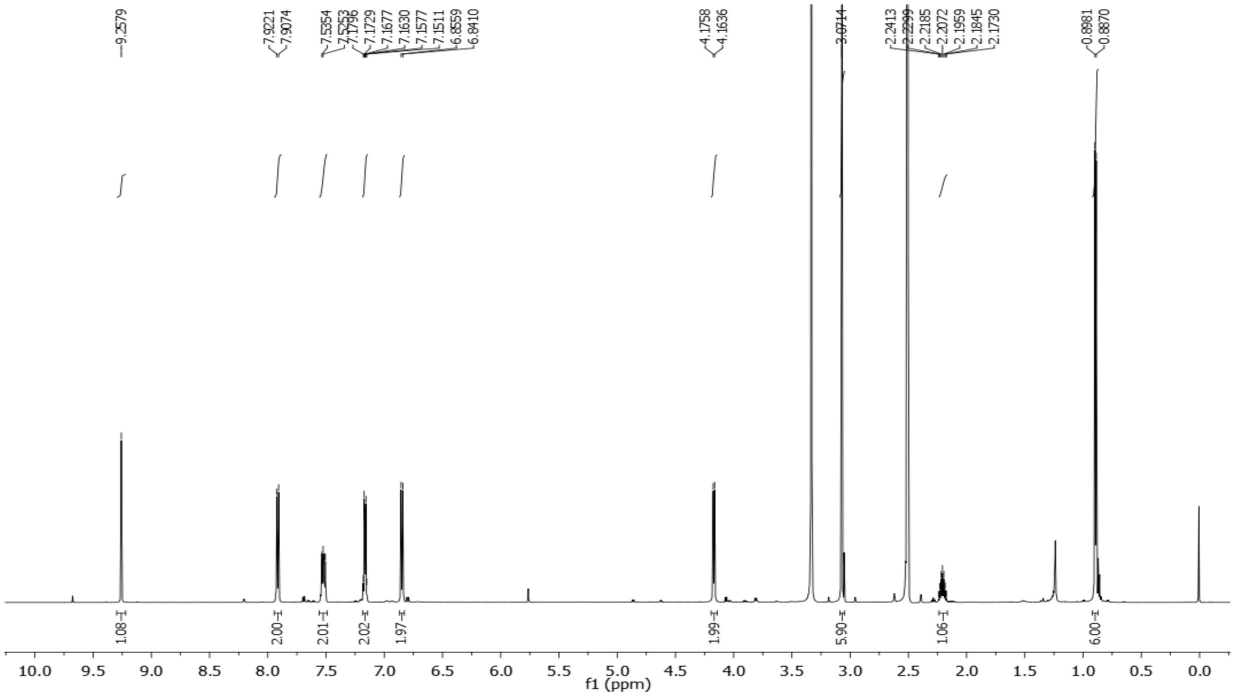

Figure S14. ^{13}C NMR spectrum (DMSO-d_6, 151 MHz) of ***(E)-4-(((1-isobutyl-1H-benzo[d]imidazol-2-yl)imino)methyl)-N,N-dimethylaniline 28***

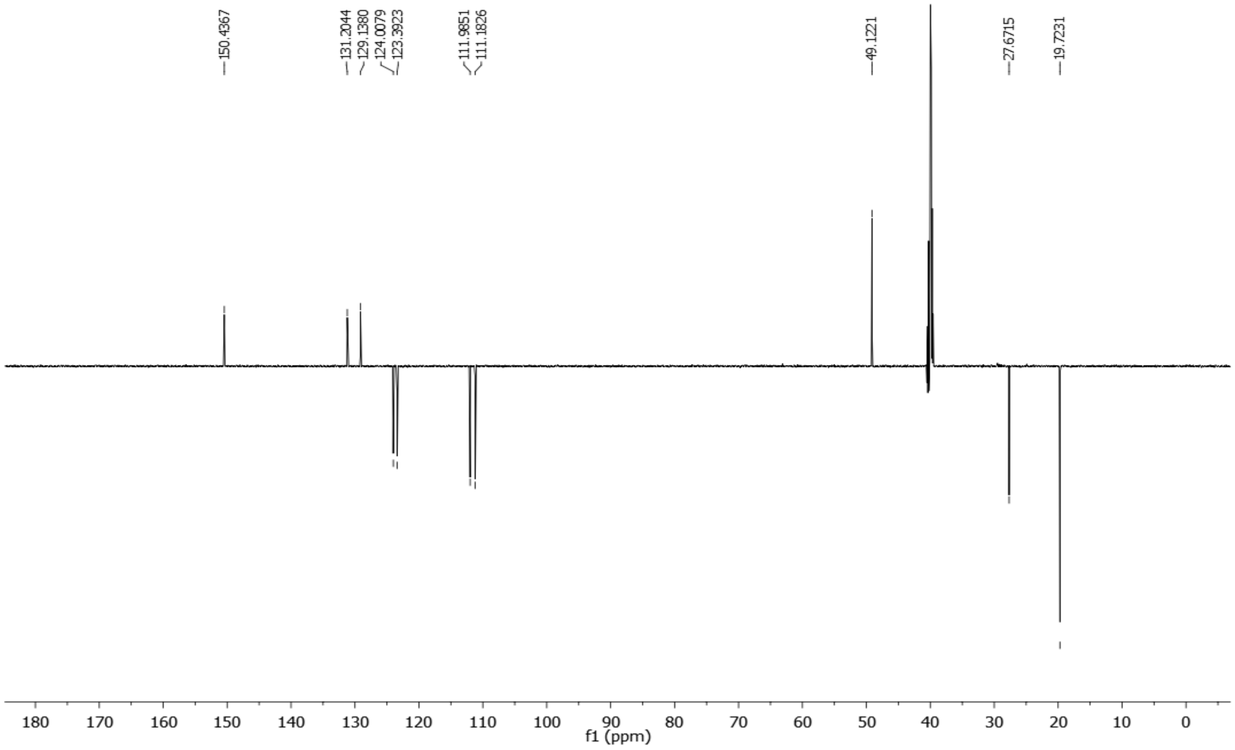

Figure S15. ^{1}H NMR spectrum (DMSO-d_6, 400 MHz) of ***E(Z)-2-((4-(dimethylamino)benzylidene)amino)-1-isobutyl-1H-benzo[d]imidazole-6-carbonitrile 29***

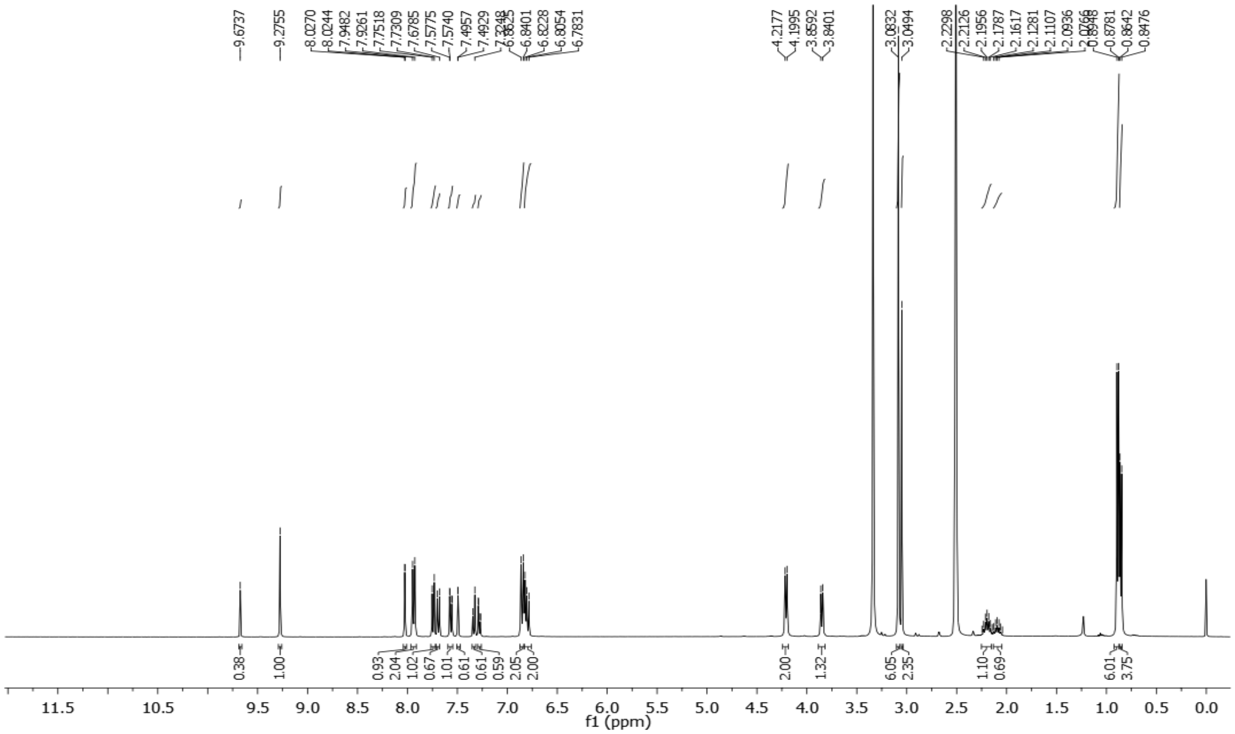

Figure S16. ^{13}C NMR spectrum (DMSO-d_6, 100 MHz) of ***E(Z)-2-((4-(dimethylamino)benzylidene)amino)-1-isobutyl-1H-benzo[d]imidazole-6-carbonitrile 29***

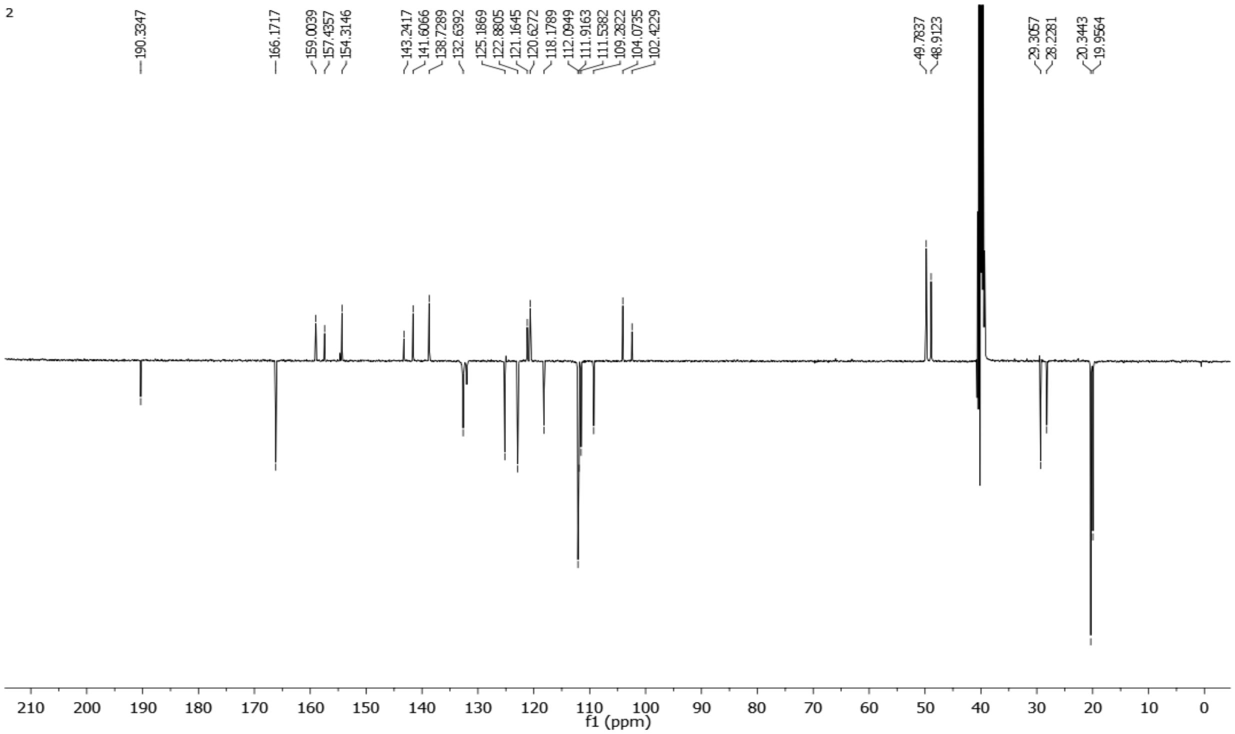

Figure S17. ^{1}H NMR spectrum (DMSO-d_6, 300 MHz) of ***(E)-5-(diethylamino)-2-(((1-isobutyl-1H-benzo[d]imidazol-2-yl)imino)methyl)phenol 30***

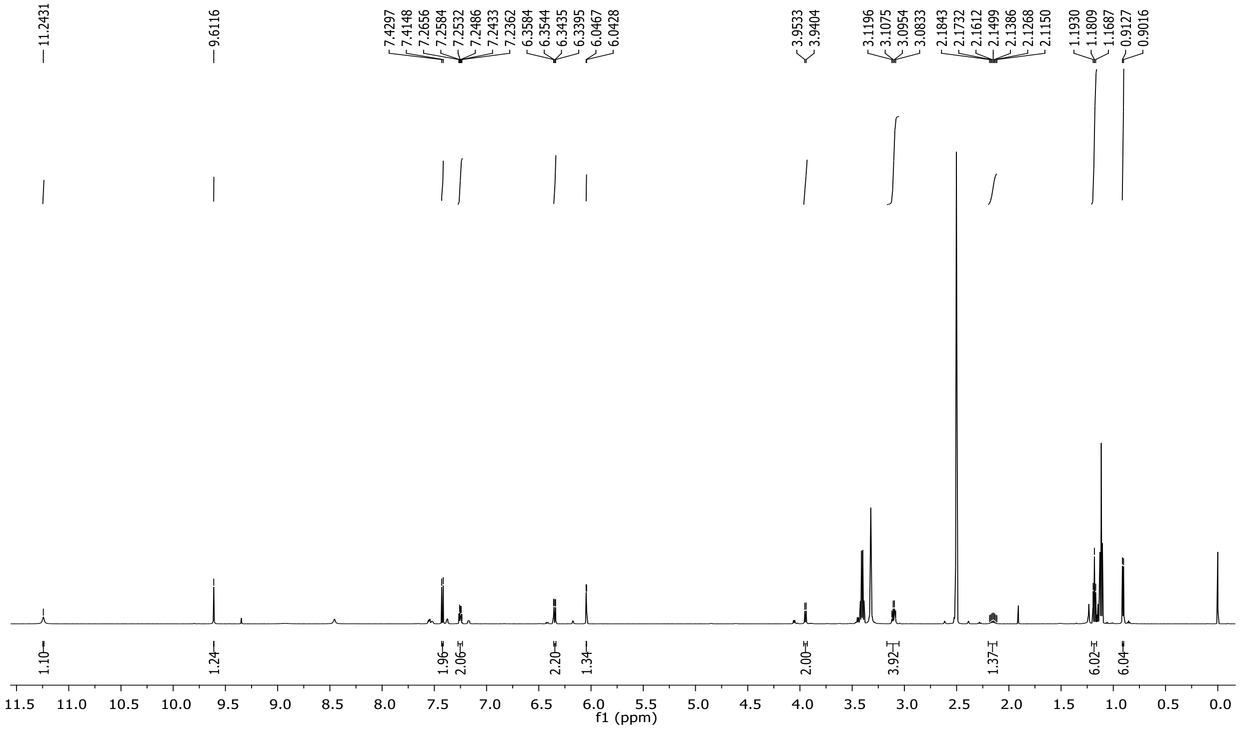

Figure S18. ^{13}C NMR spectrum (DMSO-d_6, MHz) of ***(E)-5-(diethylamino)-2-(((1-isobutyl-1H-benzo[d]imidazol-2-yl)imino)methyl)phenol 30***

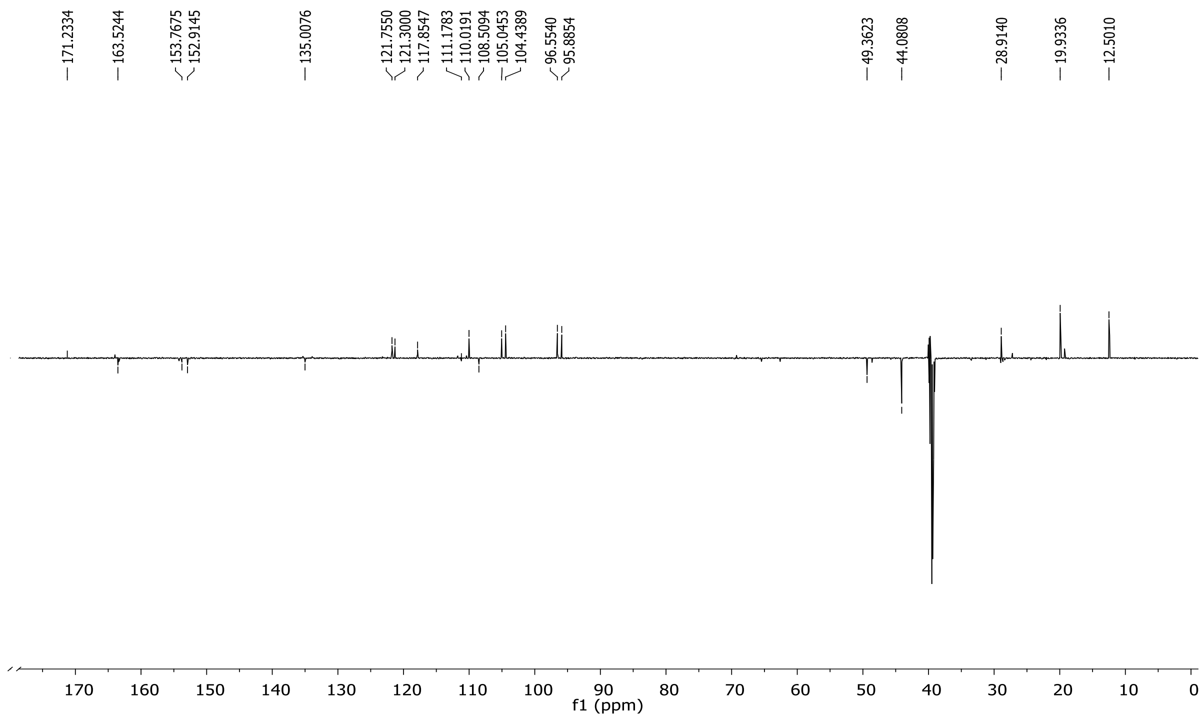

Figure S19. ^{1}H NMR spectrum (DMSO-d_6, 600 MHz) of ***(E)-2-((4-(diethylamino)-2-hydroxybenzylidene)amino)-1-isobutyl-1H-benzo[d]imidazole-6-carbonitrile 31***

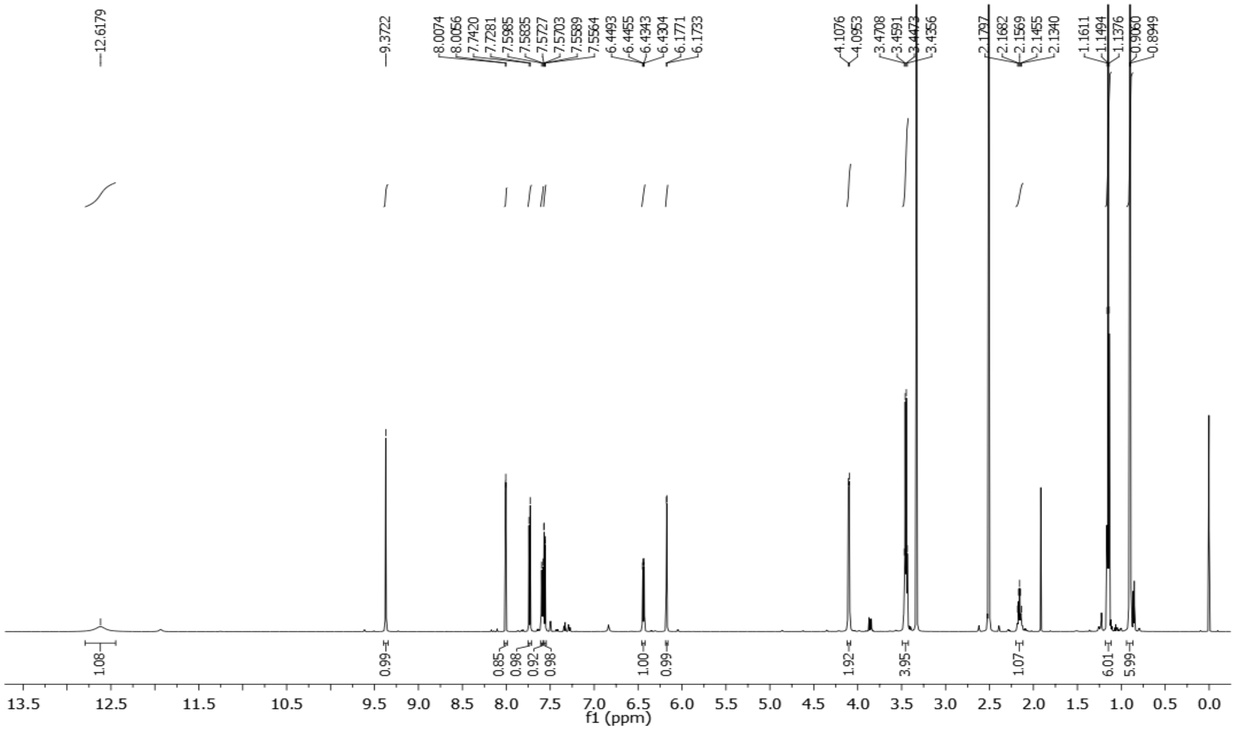

Figure S20. ^{13}C NMR spectrum (DMSO-d_6, 151 MHz) of ***(E)-2-((4-(diethylamino)-2-hydroxybenzylidene)amino)-1-isobutyl-1H-benzo[d]imidazole-6-carbonitrile 31***

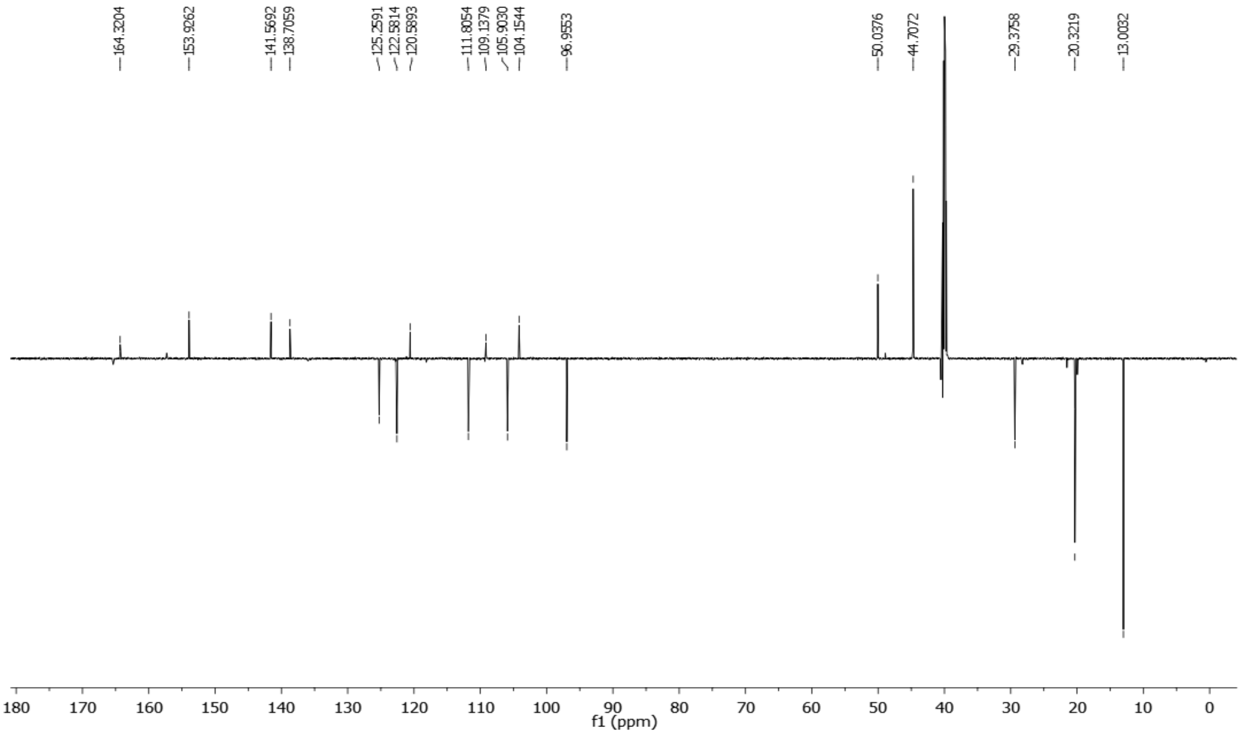

Figure S21. ^{1}H NMR spectrum (DMSO-d_6, 300 MHz) of ***(E)-1-isobutyl-2-((4-nitrobenzylidene)amino)-1H-benzo[d]imidazole-6-carbonitrile 32***

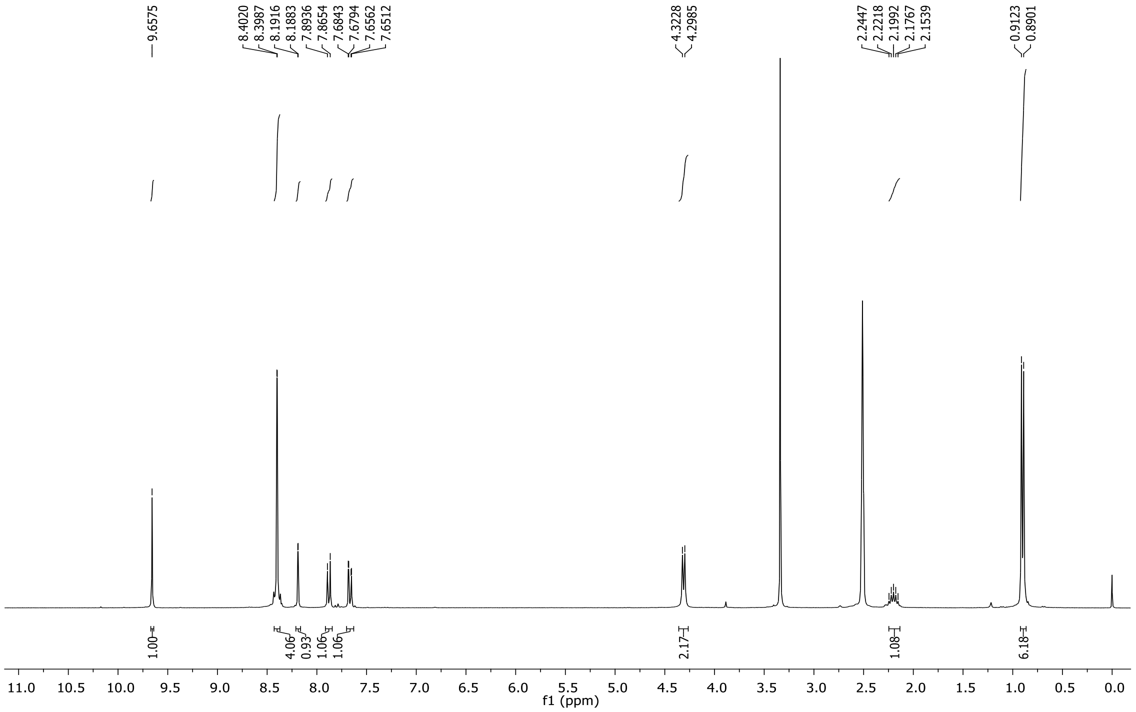

Figure S22. ^{13}C NMR spectrum (DMSO-d_6, 151 MHz) of ***(E)-1-isobutyl-2-((4-nitrobenzylidene)amino)-1H-benzo[d]imidazole-6-carbonitrile 32***

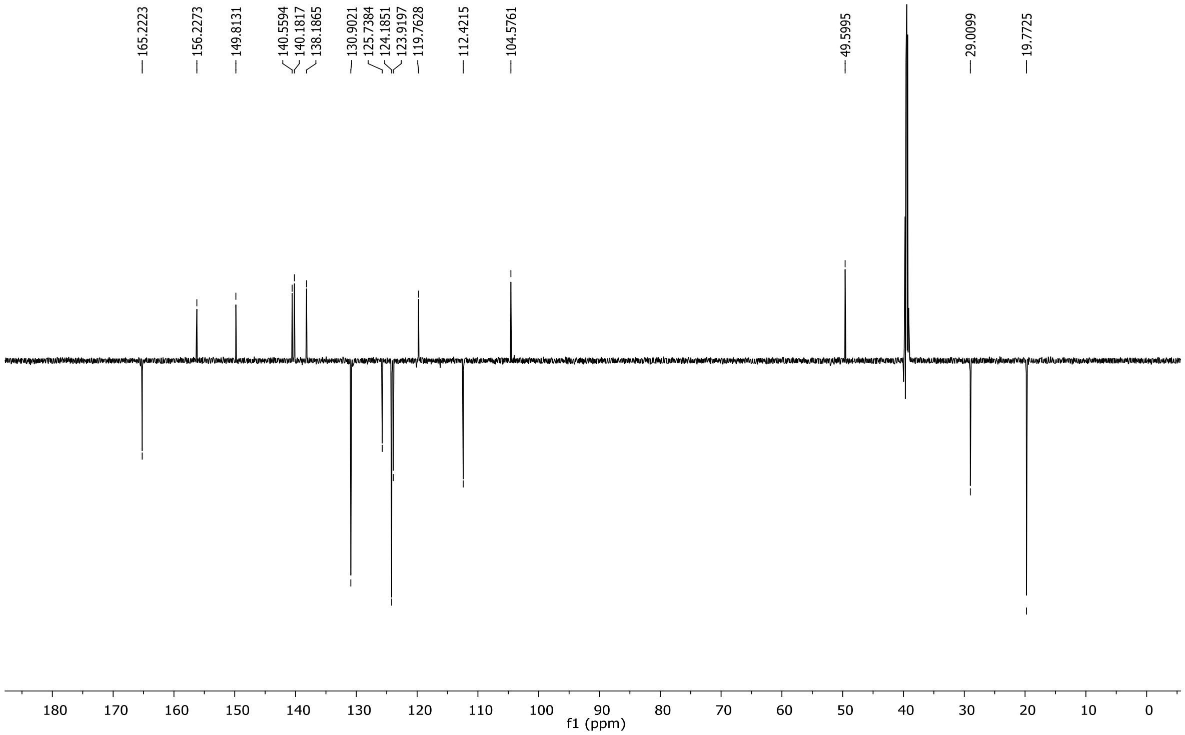

Figure S23. ^{1}H NMR spectrum (DMSO-d_6, 600 MHz) of ***(E)-N,N-dimethyl-4-(((1-methyl-1H-benzo[d]imidazol-2-yl)imino)methyl)aniline 33***

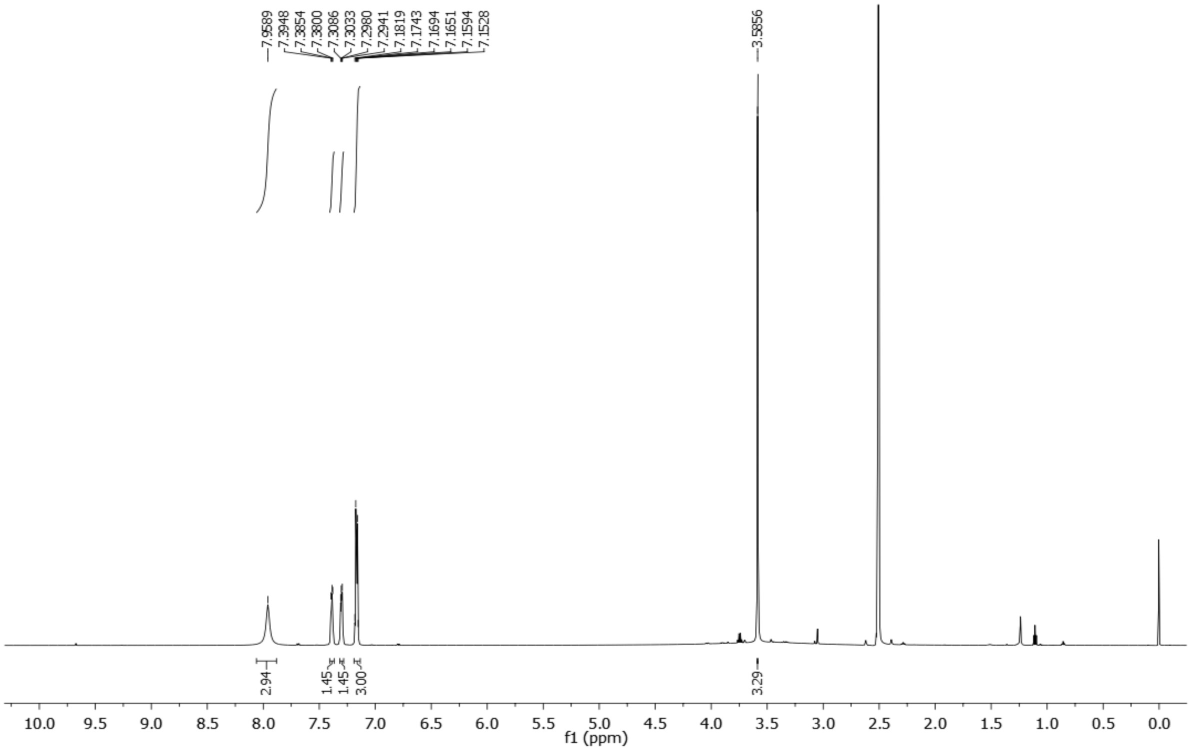

Figure S24. ^{13}C NMR spectrum (DMSO-d_6, MHz) of ***(E)-N,N-dimethyl-4-(((1-methyl-1H-benzo[d]imidazol-2-yl)imino)methyl)aniline 33***

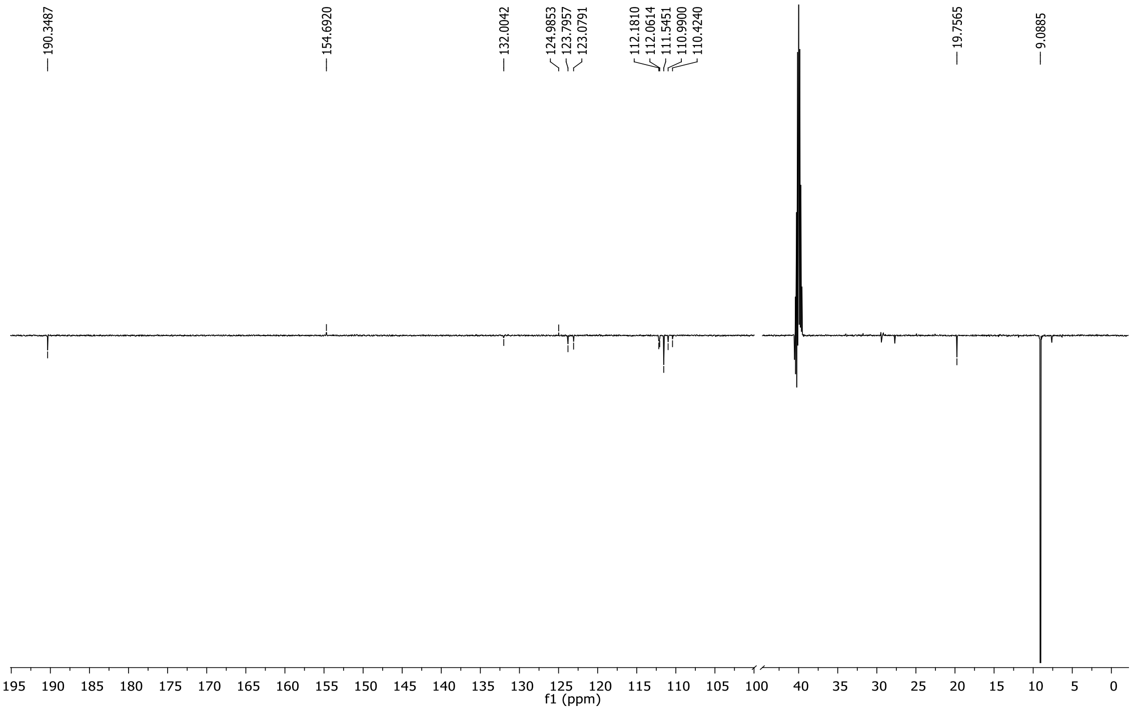

Figure S25. ^{1}H NMR spectrum (DMSO-d_6, 400 MHz) of ***(E)-2-((4-(dimethylamino)benzylidene)amino)-1-methyl-1H-benzo[d]imidazole-6-carbonitrile 34***

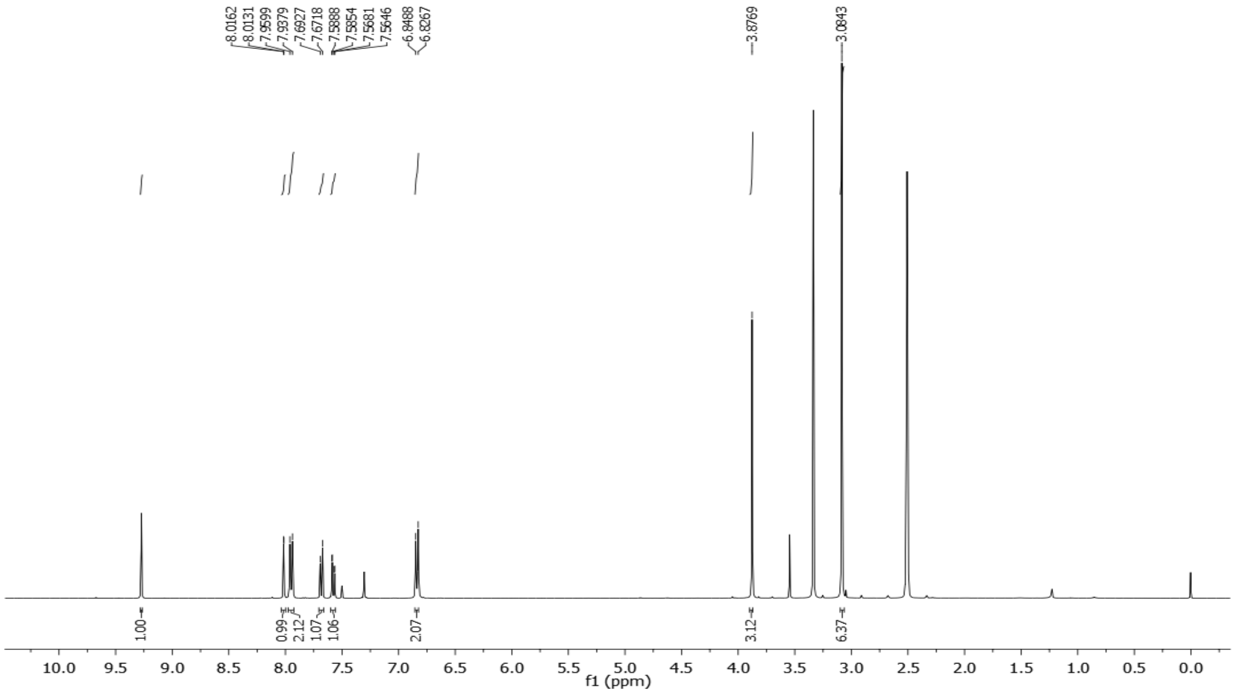

Figure S26. ^{13}C NMR spectrum (DMSO-d_6, 100 MHz) of ***(E)-2-((4-(dimethylamino)benzylidene)amino)-1-methyl-1H-benzo[d]imidazole-6-carbonitrile 34***

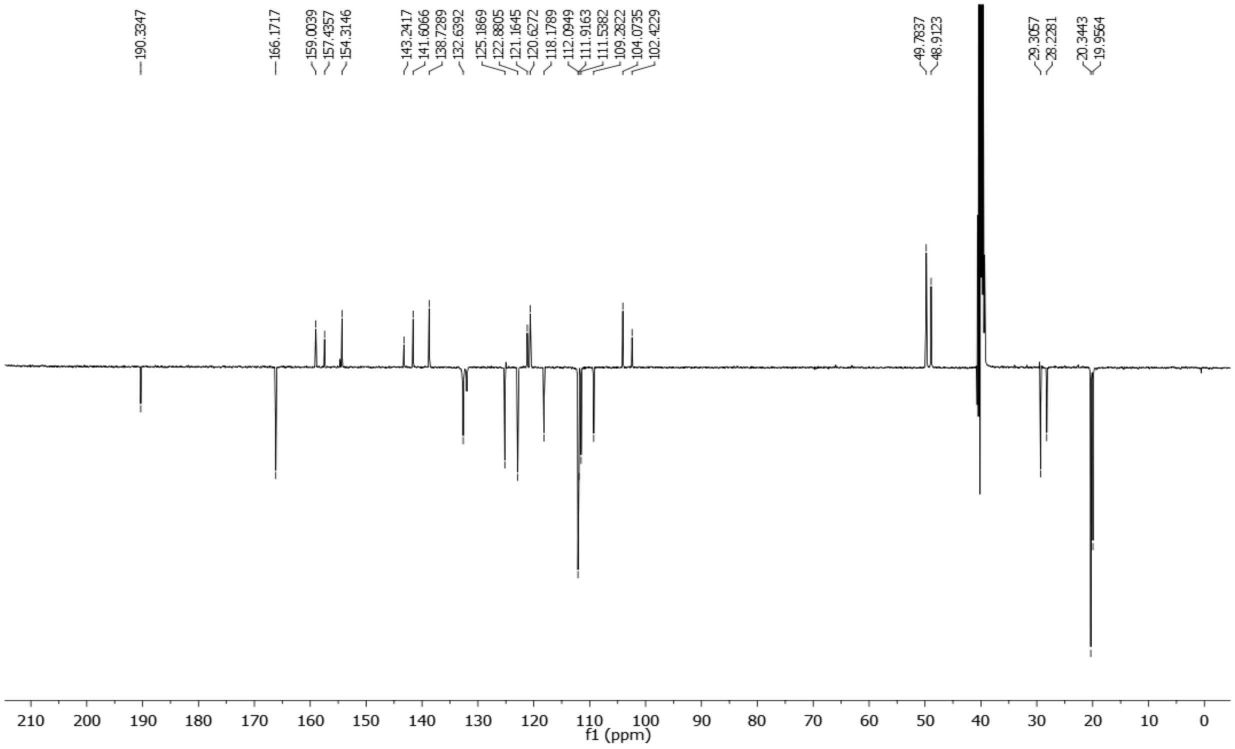

Figure S27. ^{1}H NMR spectrum (DMSO-d_6, 600 MHz) of ***(E)-5-(diethylamino)-2-(((1-methyl-1H-benzo[d]imidazol-2-yl)imino)methyl)phenol*** **35**

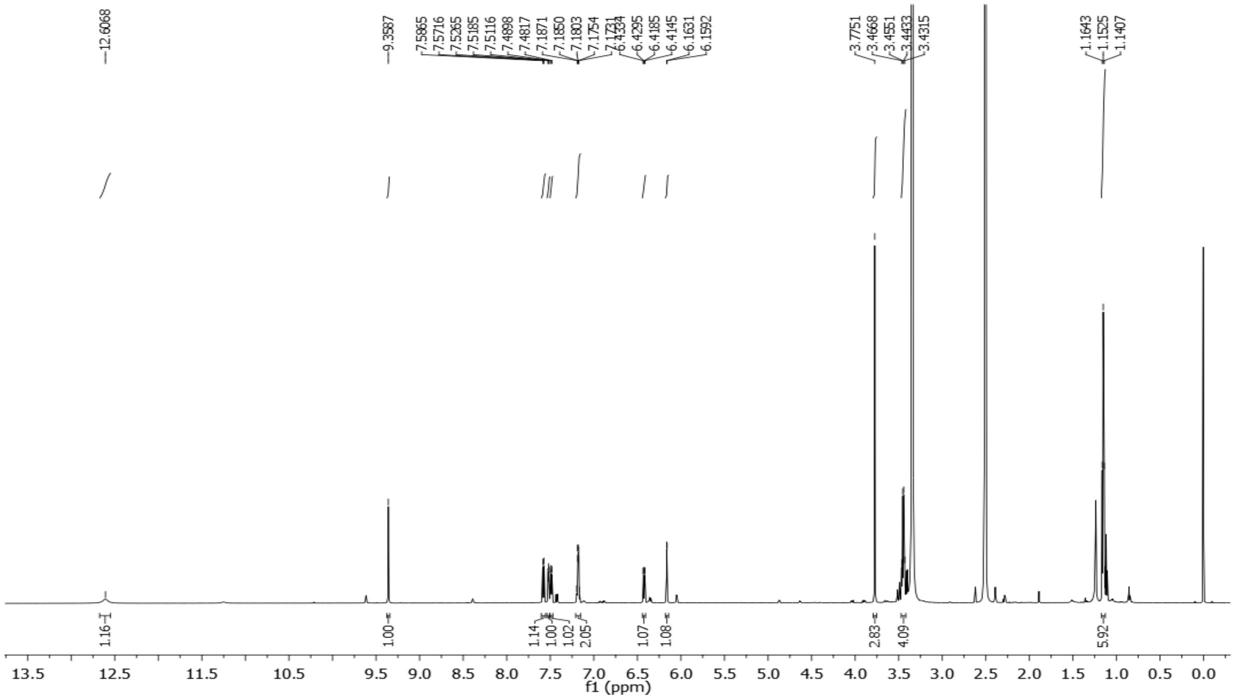

Figure S28. ^{13}C NMR spectrum (DMSO-d_6, 151 MHz) of ***(E)-5-(diethylamino)-2-(((1-methyl-1H-benzo[d]imidazol-2-yl)imino)methyl)phenol*** **35**

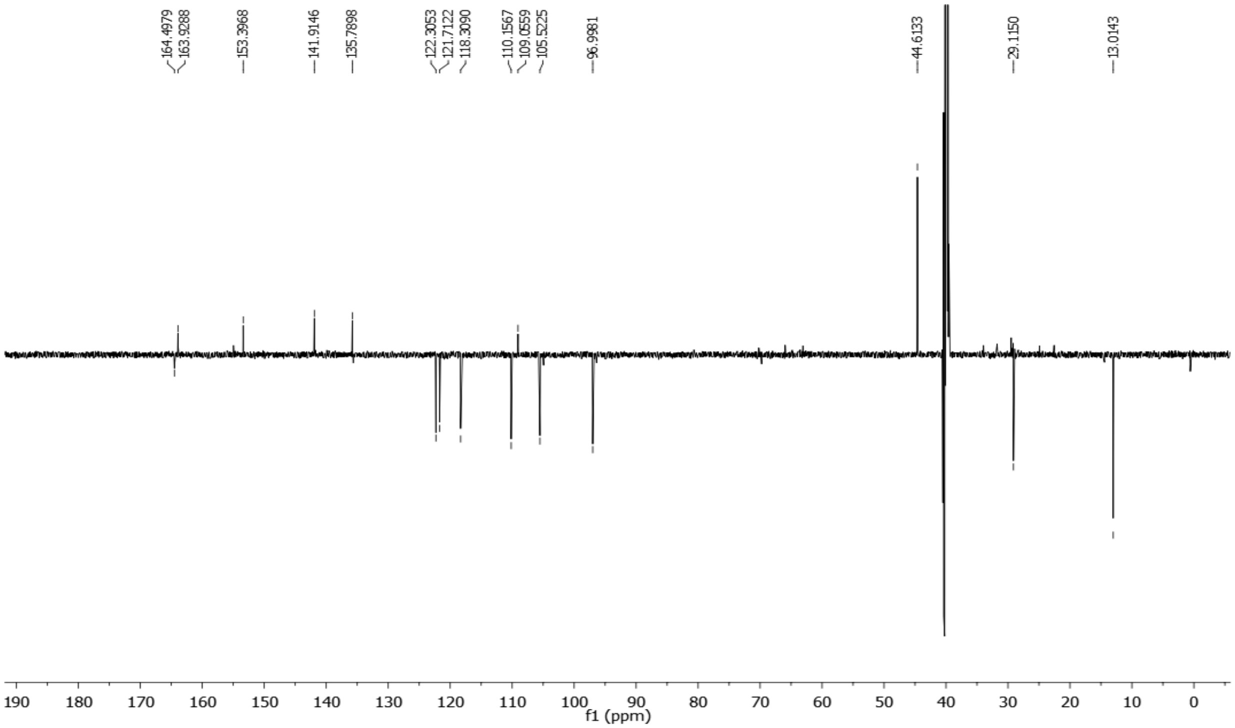

Figure S29. ^{1}H NMR spectrum (DMSO-d_6, 400 MHz) of ***(E)-2-((4-(diethylamino)-2-hydroxybenzylidene)amino)-1-methyl-1H-benzo[d]imidazole-6-carbonitrile 36***

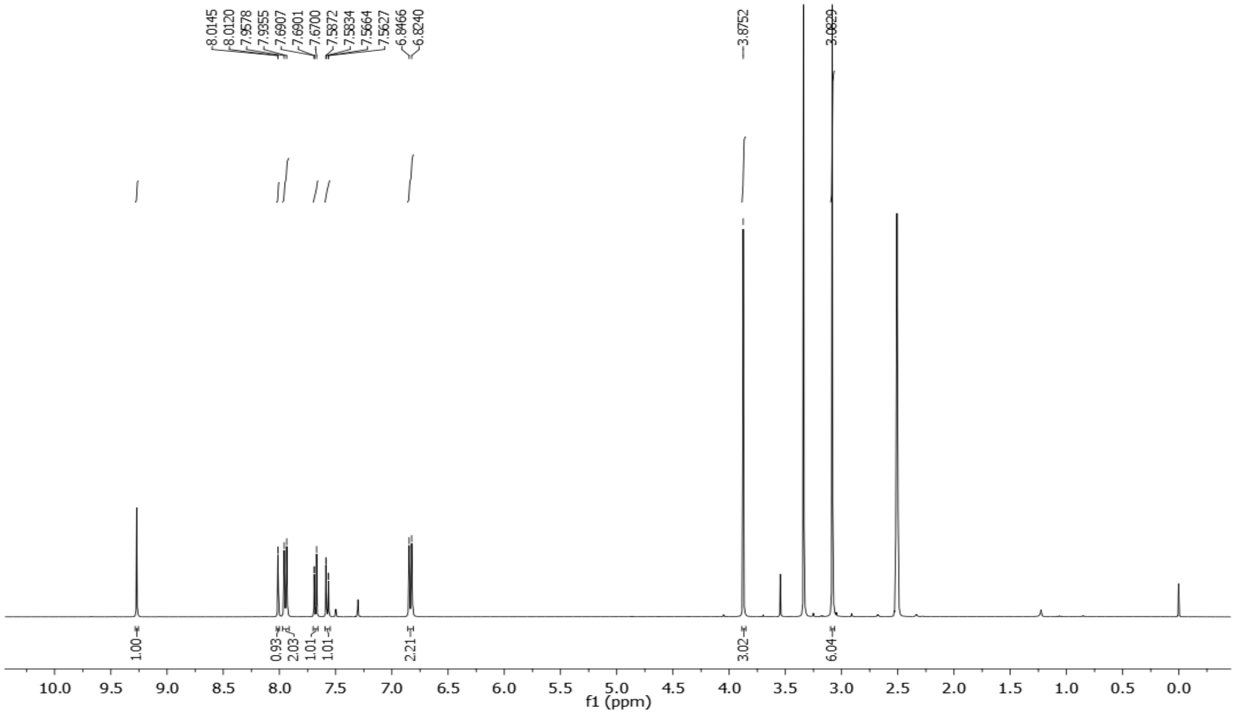

Figure S30. ^{13}C NMR spectrum (DMSO-d_6, 100 MHz) of ***(E)-2-((4-(diethylamino)-2-hydroxybenzylidene)amino)-1-methyl-1H-benzo[d]imidazole-6-carbonitrile 36***

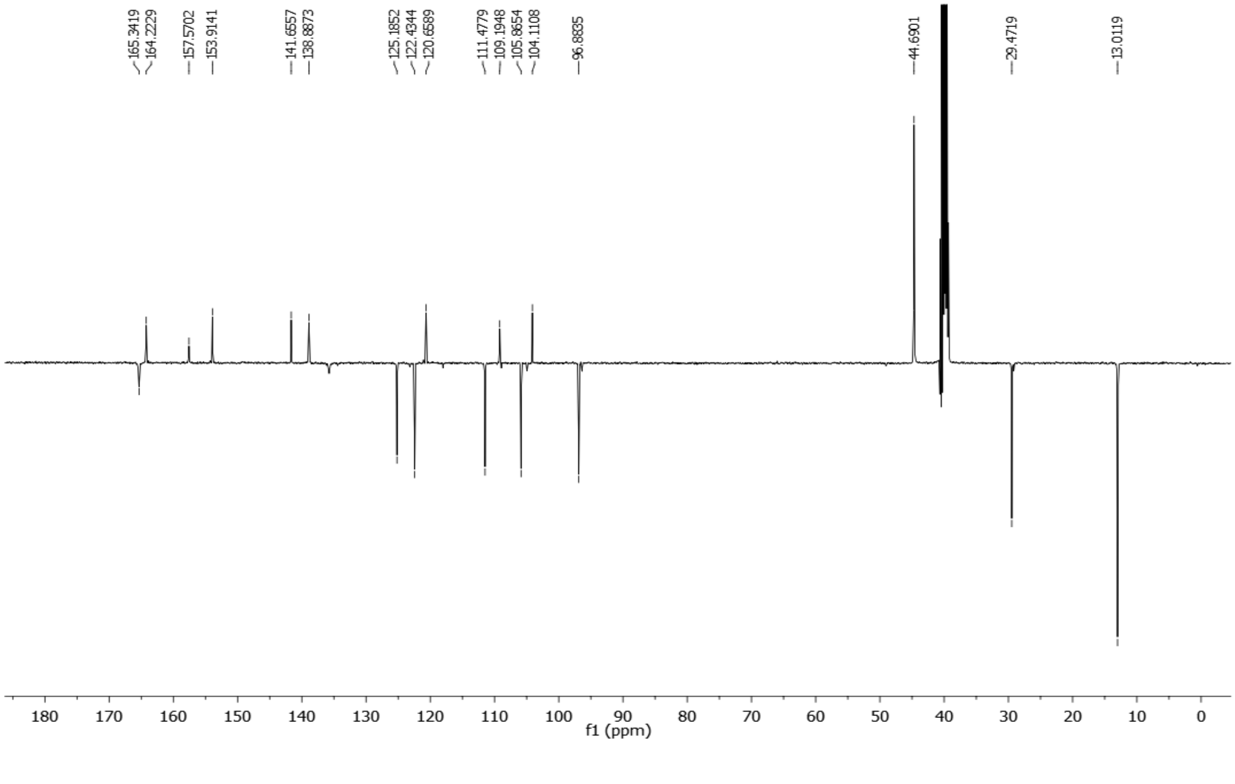

Figure S31. ^{1}H NMR spectrum (DMSO-d_6, 400 MHz) of ***(E)-1-methyl-2-((4-nitrobenzylidene)amino)-1H-benzo[d]imidazole-6-carbonitrile 37***

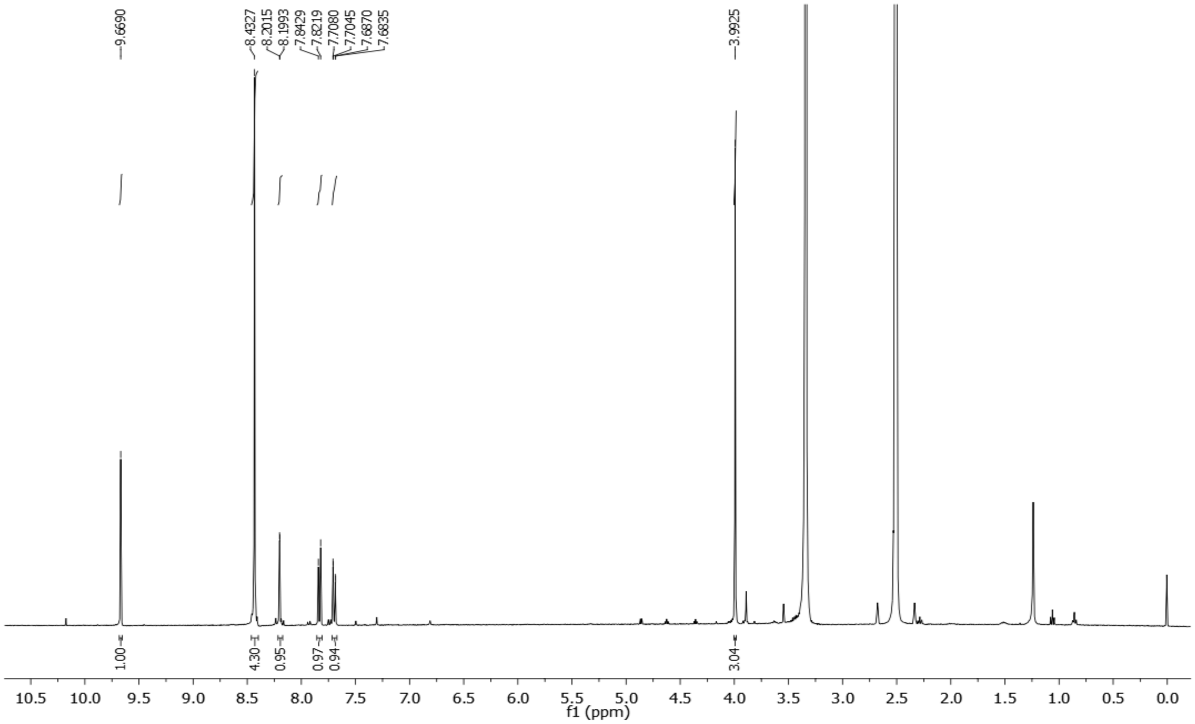

Figure S32. ^{13}C NMR spectrum (DMSO-d_6, MHz) of ***(E)-1-methyl-2-((4-nitrobenzylidene)amino)-1H-benzo[d]imidazole-6-carbonitrile 37***

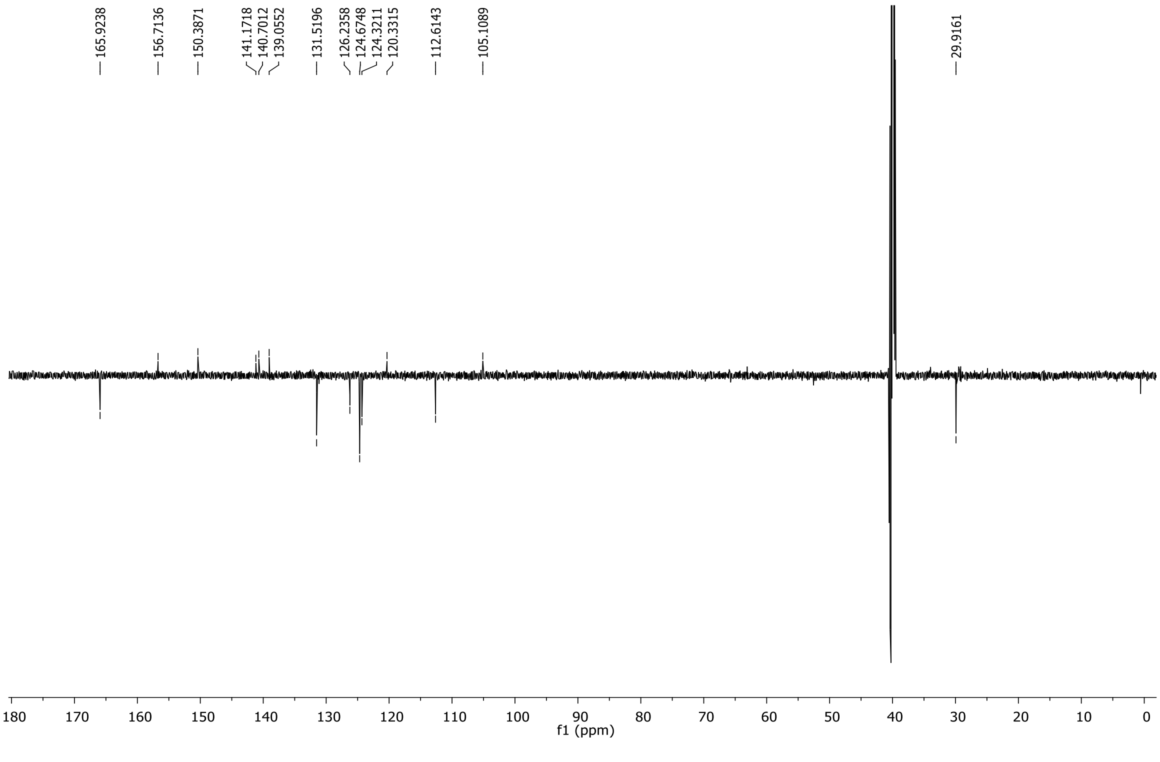

Figure S33. ^{1}H NMR spectrum (DMSO-d_6, 400 MHz) of ***(E)-N,N-dimethyl-4-(((1-phenyl-1H-benzo[d]imidazol-2-yl)imino)methyl)aniline* 38**

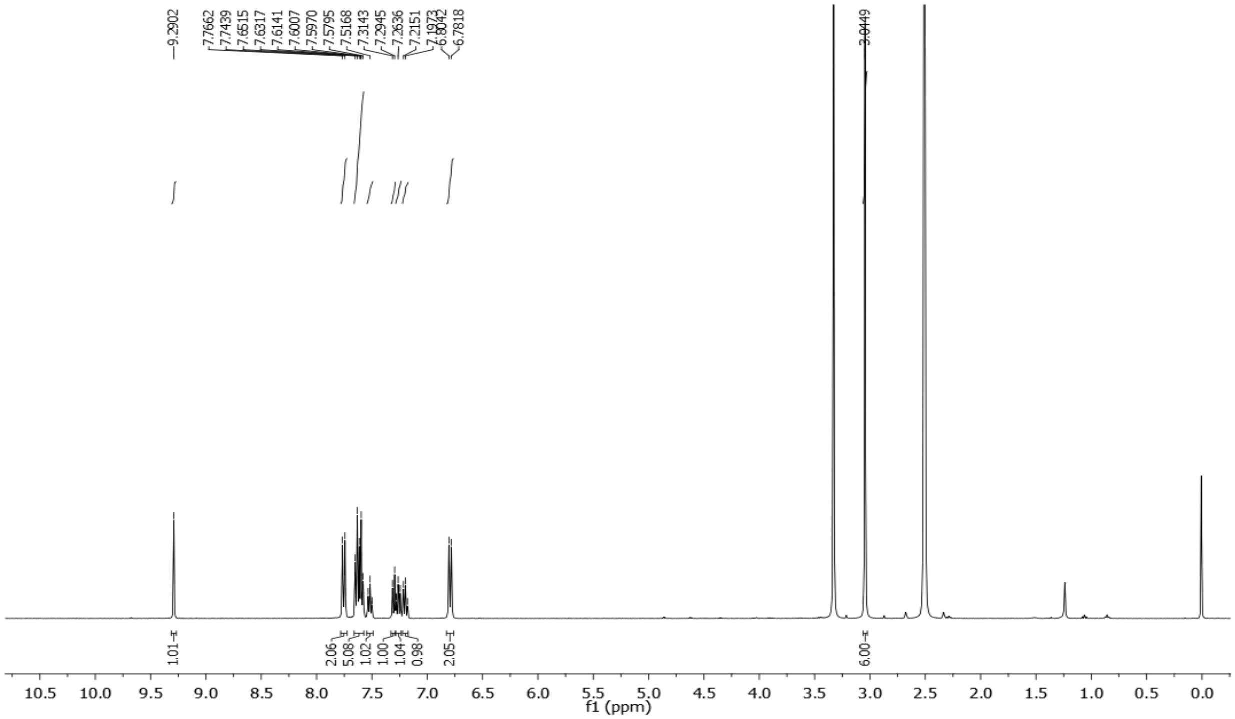

Figure S34. ^{13}C NMR spectrum (DMSO-d_6, 151 MHz) of ***(E)-N,N-dimethyl-4-(((1-phenyl-1H-benzo[d]imidazol-2-yl)imino)methyl)aniline* 38**

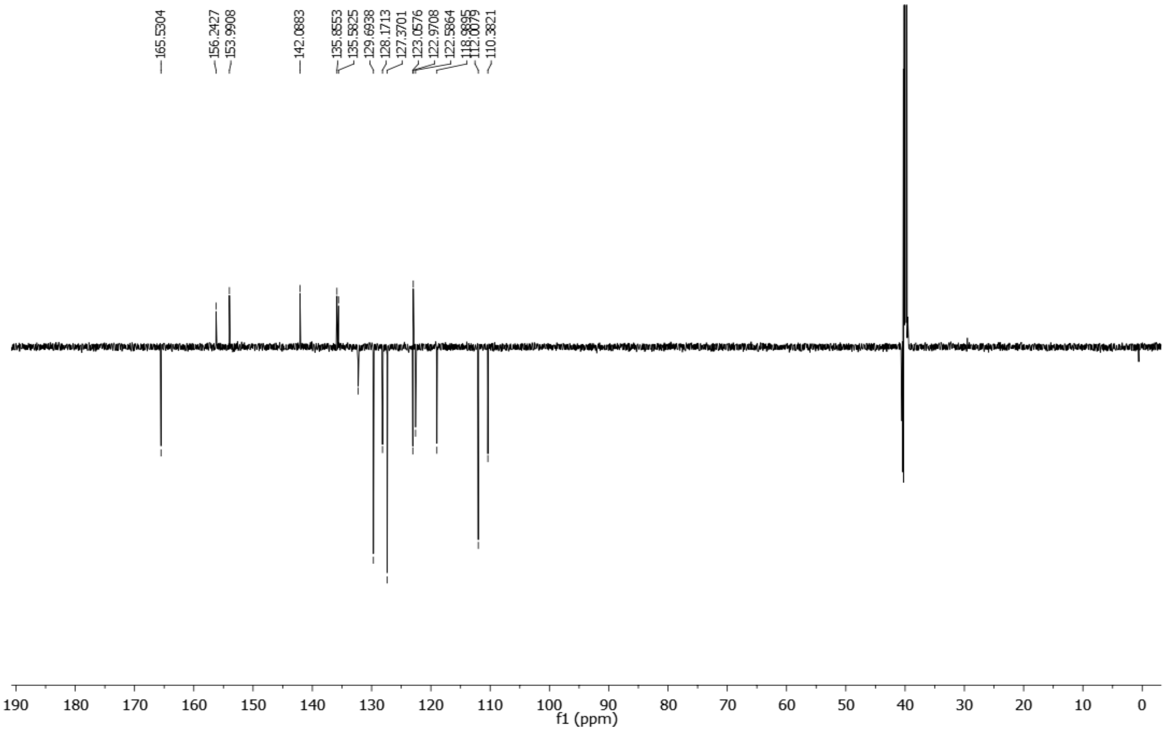

Figure S35. ^{1}H NMR spectrum (DMSO-d_6, 600 MHz) of ***(E)-2-((4-(dimethylamino)benzylidene)amino)-1-phenyl-1H-benzo[d]imidazole-6-carbonitrile 39***

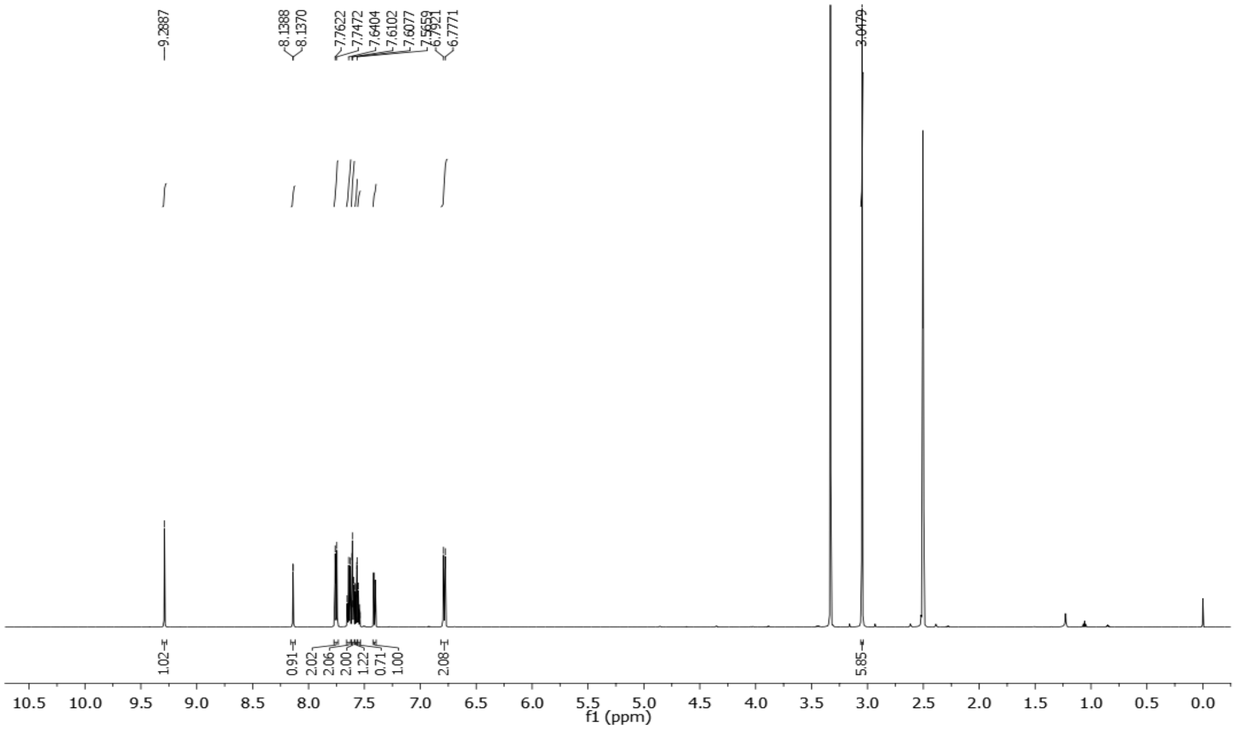

Figure S36. ^{13}C NMR spectrum (DMSO-d_6, 151 MHz) of ***(E)-2-((4-(dimethylamino)benzylidene)amino)-1-phenyl-1H-benzo[d]imidazole-6-carbonitrile 39***

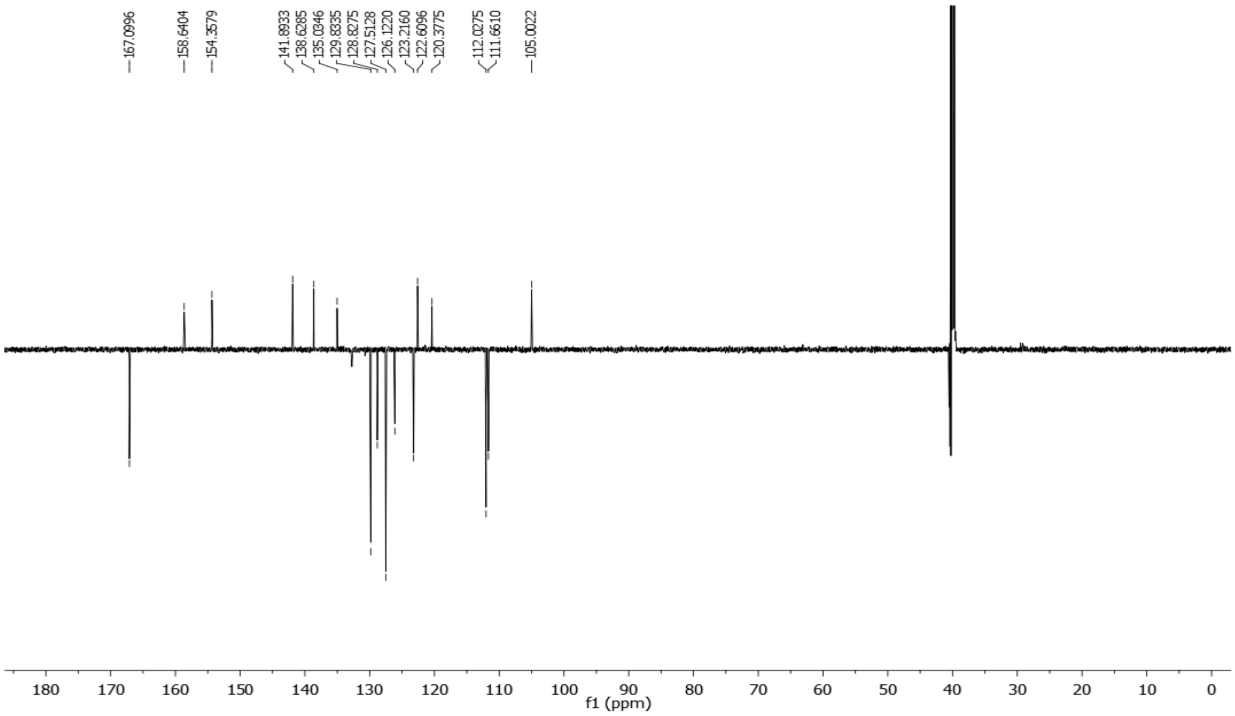

Figure S37. ^{1}H NMR spectrum (DMSO-d_6, 400 MHz) of ***(E)-5-(diethylamino)-2-(((1-phenyl-1H-benzo[d]imidazol-2-yl)imino)methyl)phenol*** ***40***

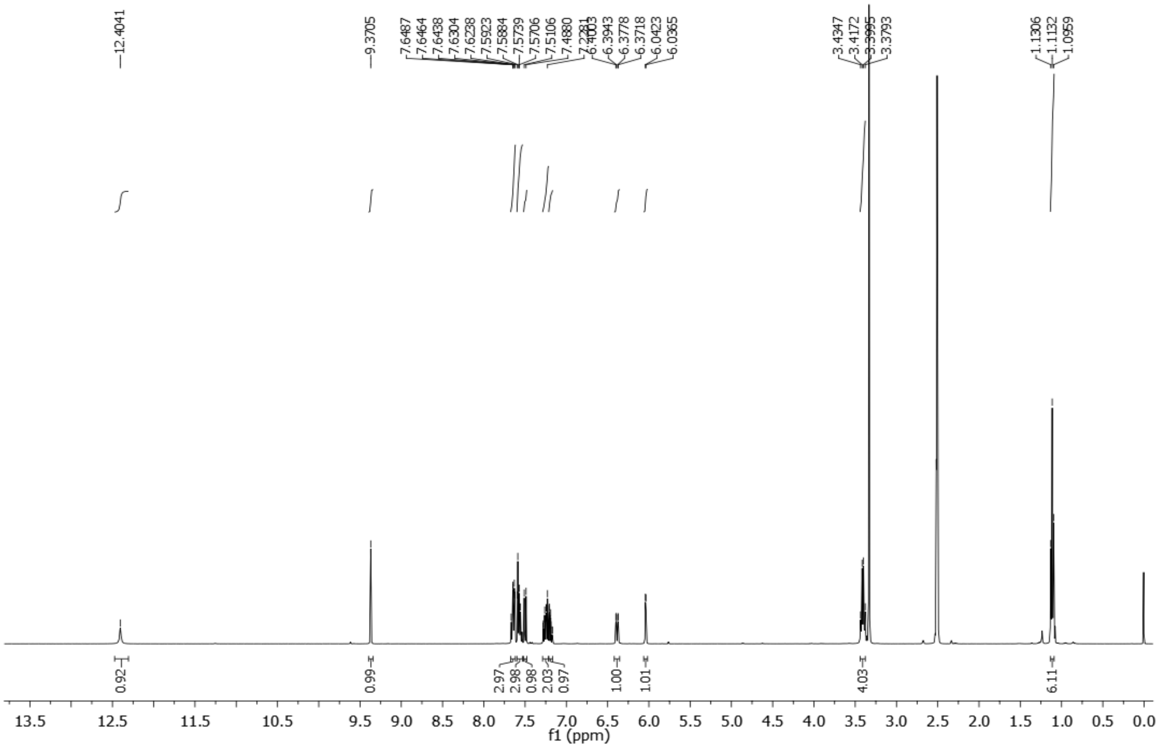

Figure S38. ^{13}C NMR spectrum (DMSO-d_6, 75 MHz) of ***(E)-5-(diethylamino)-2-(((1-phenyl-1H-benzo[d]imidazol-2-yl)imino)methyl)phenol 40***

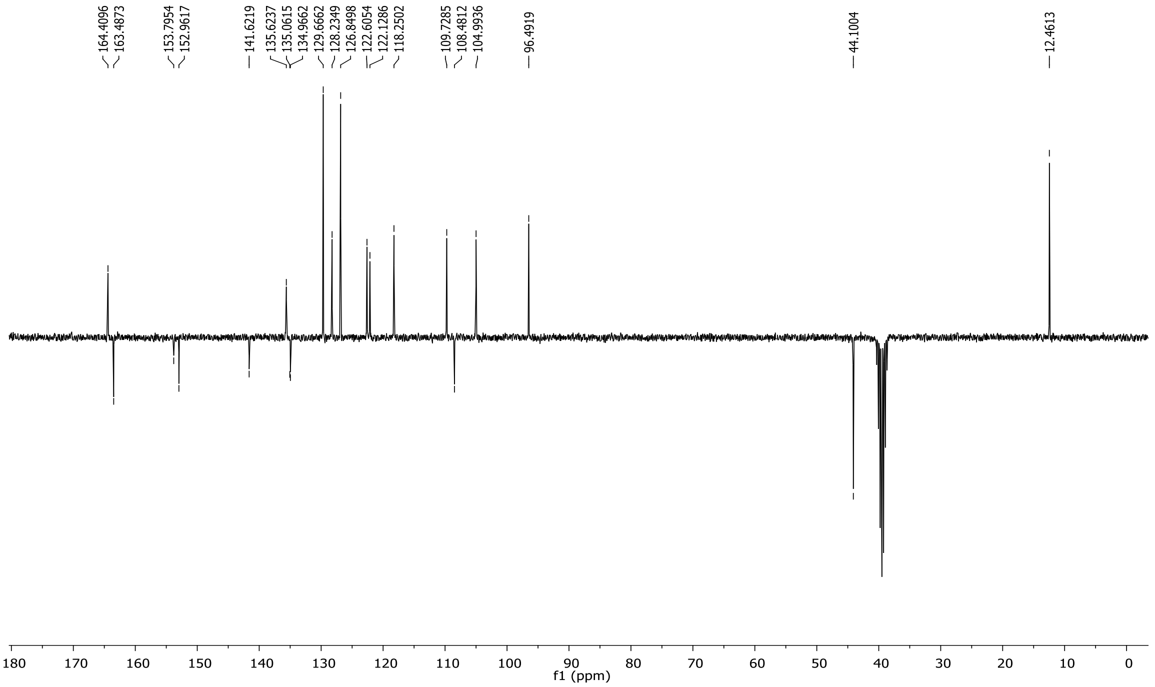

Figure S39. ^{1}H NMR spectrum (DMSO-d_6, 400 MHz) of ***(E)-2-((4-(diethylamino)-2-hydroxybenzylidene)amino)-1-phenyl-1H-benzo[d]imidazole-6-carbonitrile 41***

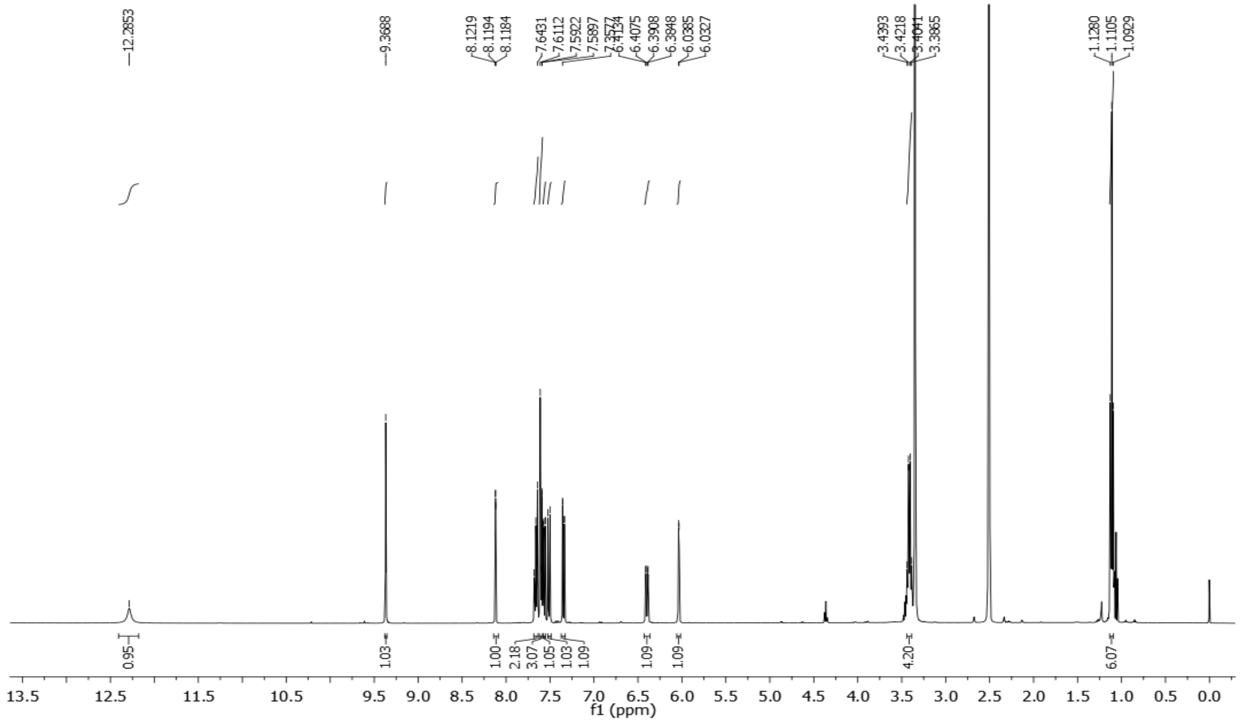

Figure S40. ^{13}C NMR spectrum (DMSO-d_6, 100 MHz) of ***(E)-2-((4-(diethylamino)-2-hydroxybenzylidene)amino)-1-phenyl-1H-benzo[d]imidazole-6-carbonitrile 41***

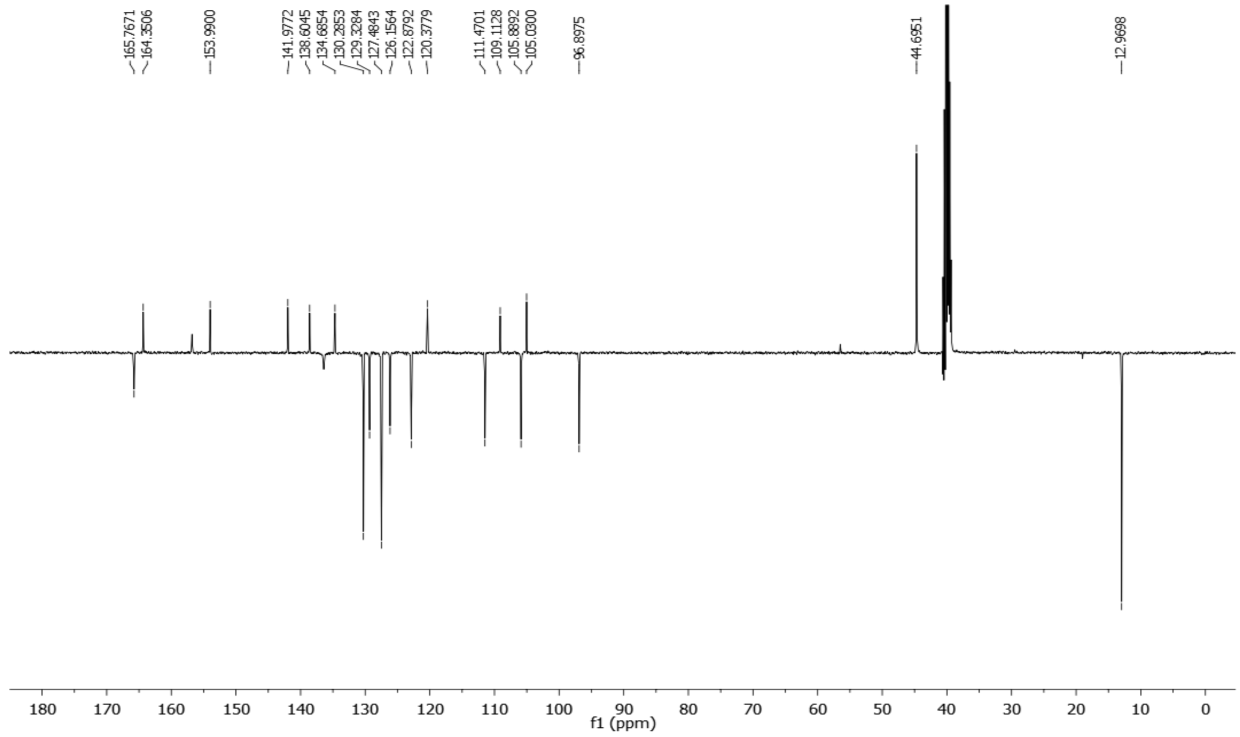

Figure S41. ^{1}H NMR spectrum (DMSO-d_6, 600 MHz) of ***E(Z)-4-(((1-hexyl-1H-benzo[d]imidazol-2-yl)imino)methyl)-N,N-dimethylaniline*** ***42***

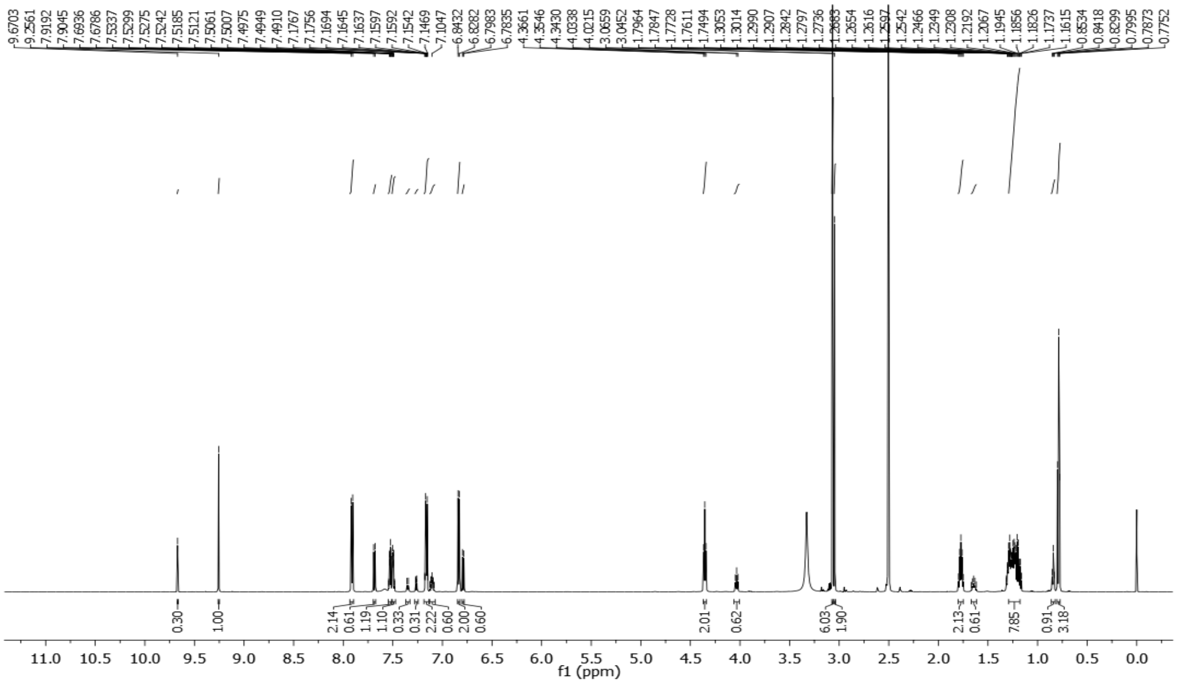

Figure S42. ^{13}C NMR spectrum (DMSO-d_6, MHz) of ***E(Z)-4-(((1-hexyl-1H-benzo[d]imidazol-2-yl)imino)methyl)-N,N-dimethylaniline*** ***42***

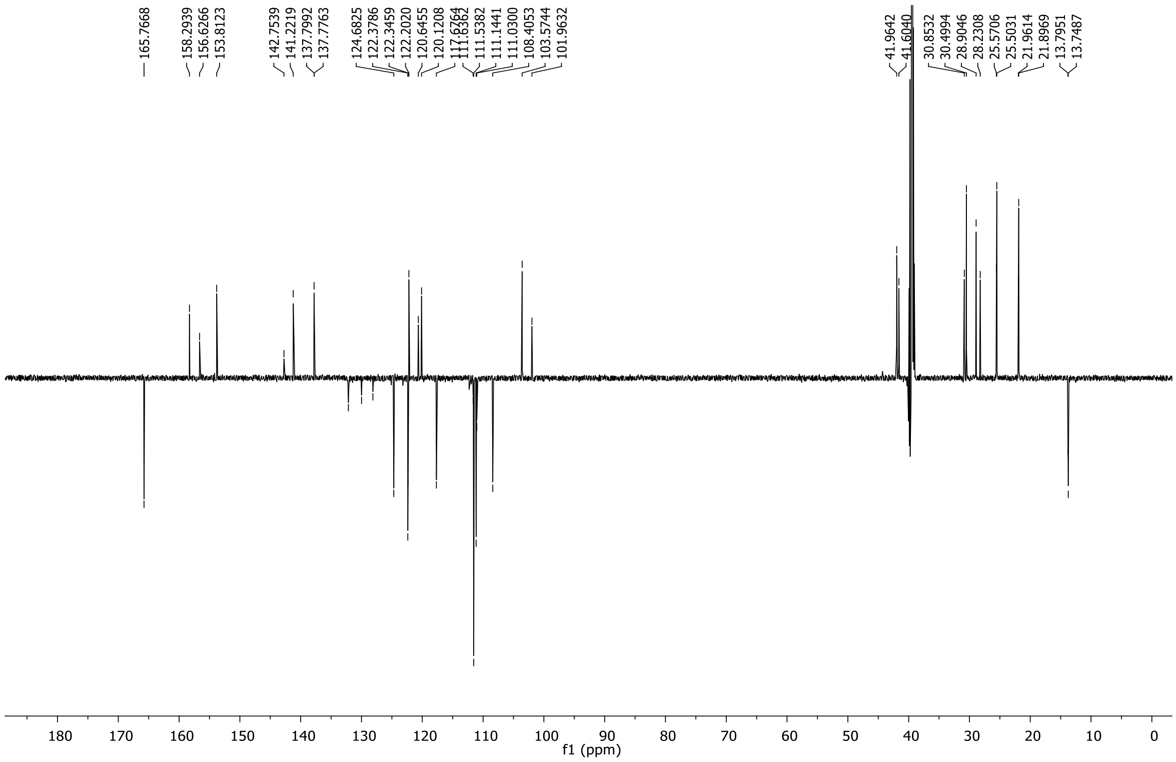

Figure S43. ^{1}H NMR spectrum (DMSO-d_6, 300 MHz) of ***(E)-2-((4-(dimethylamino)benzylidene)amino)-1-hexyl-1H-benzo[d]imidazole-6-carbonitrile 43***

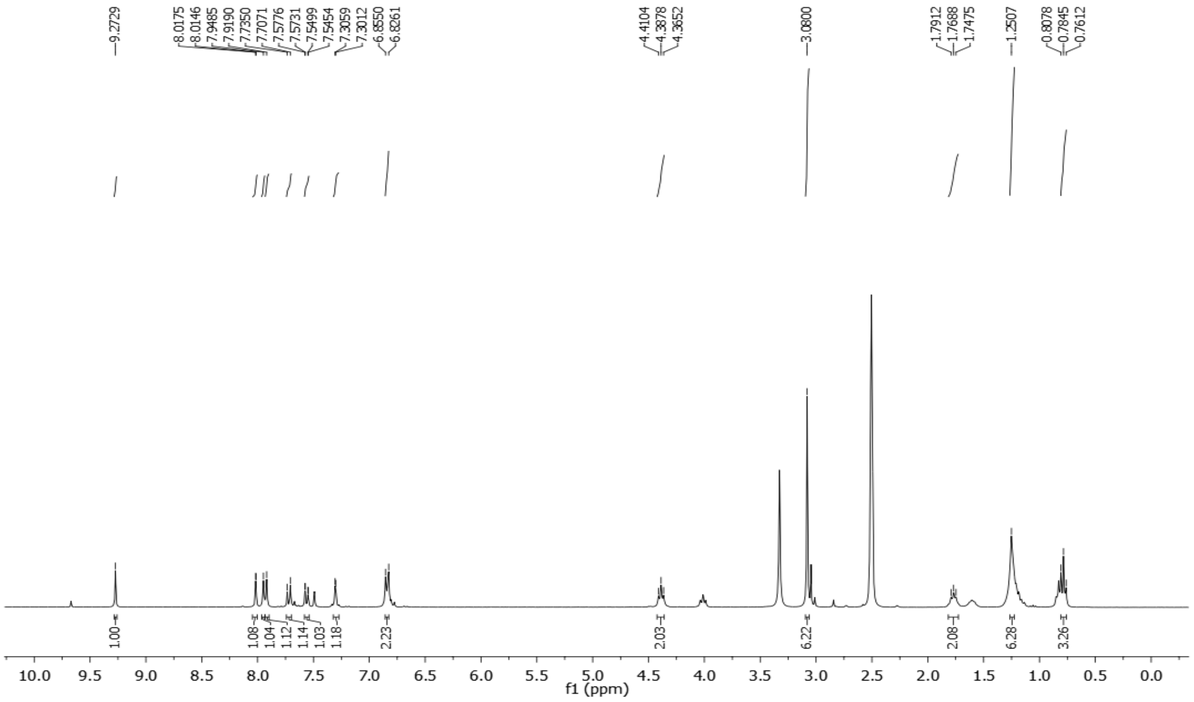

Figure S44. ^{13}C NMR spectrum (DMSO-d_6, MHz) of ***(E)-2-((4-(dimethylamino)benzylidene)amino)-1-hexyl-1H-benzo[d]imidazole-6-carbonitrile 43***

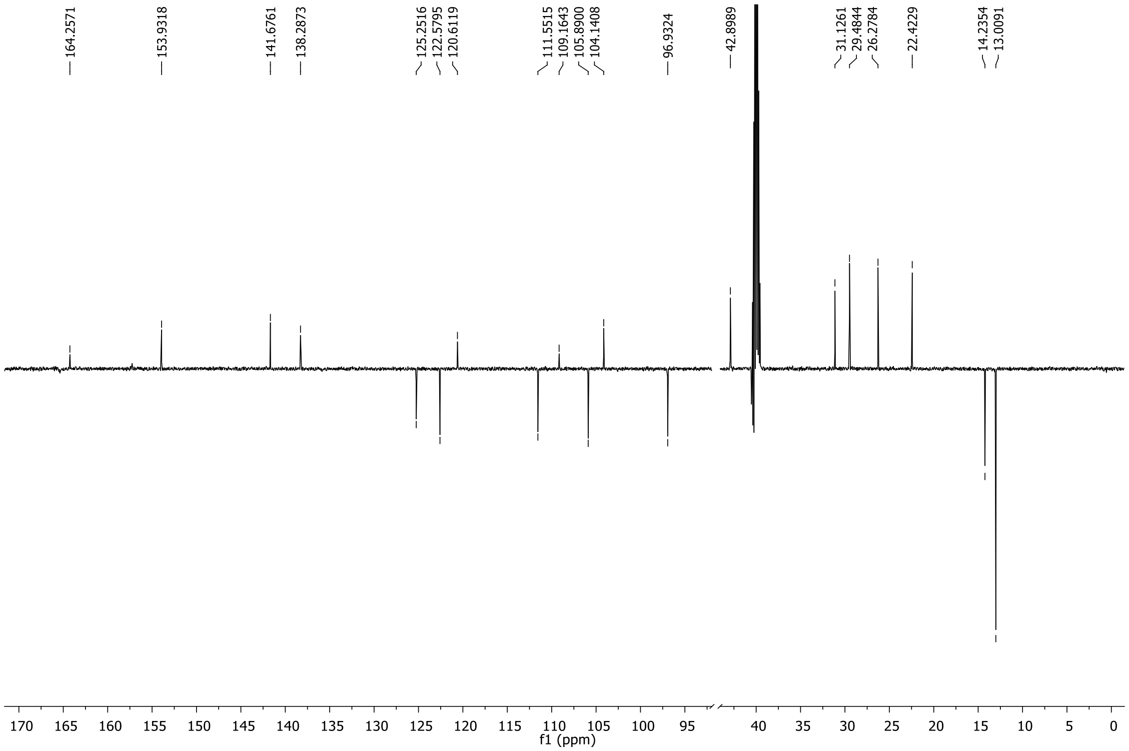

Figure S45. ^{1}H NMR spectrum (DMSO-d_6, 600 MHz) of ***(E)-5-(diethylamino)-2-(((1-hexyl-1H-benzo[d]imidazol-2-yl)imino)methyl)phenol*** ***44***

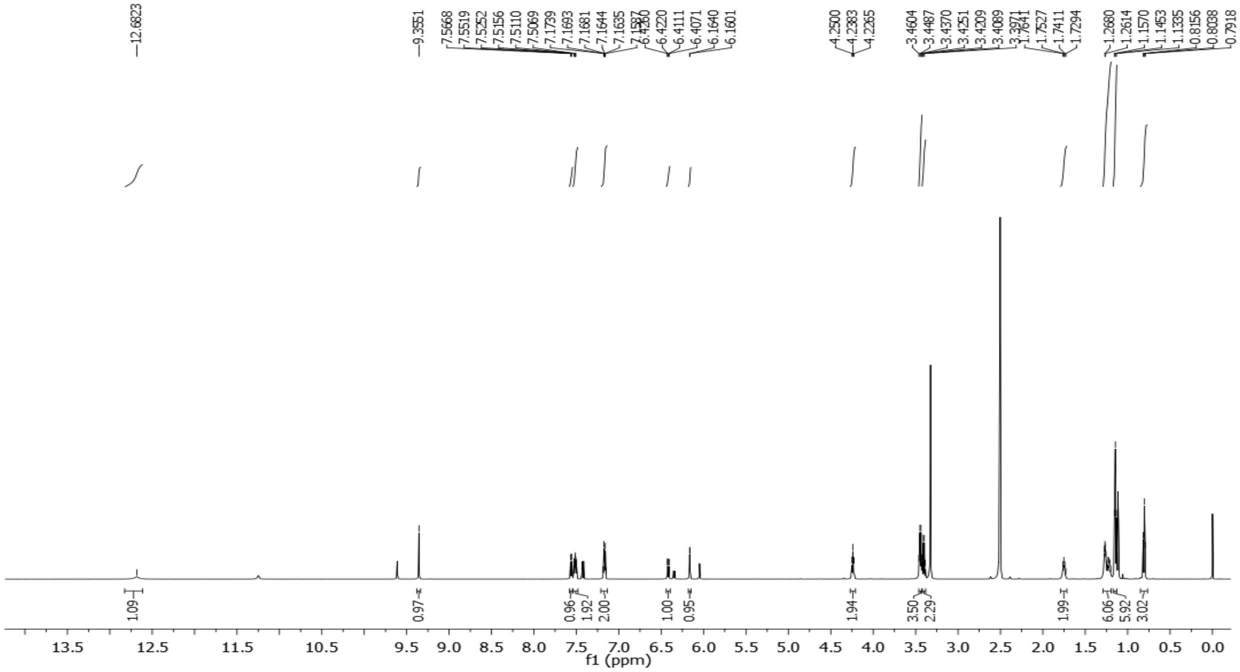

Figure S46. ^{13}C NMR spectrum (DMSO-d_6, 100 MHz) of ***(E)-5-(diethylamino)-2-(((1-hexyl-1H-benzo[d]imidazol-2-yl)imino)methyl)phenol 44***

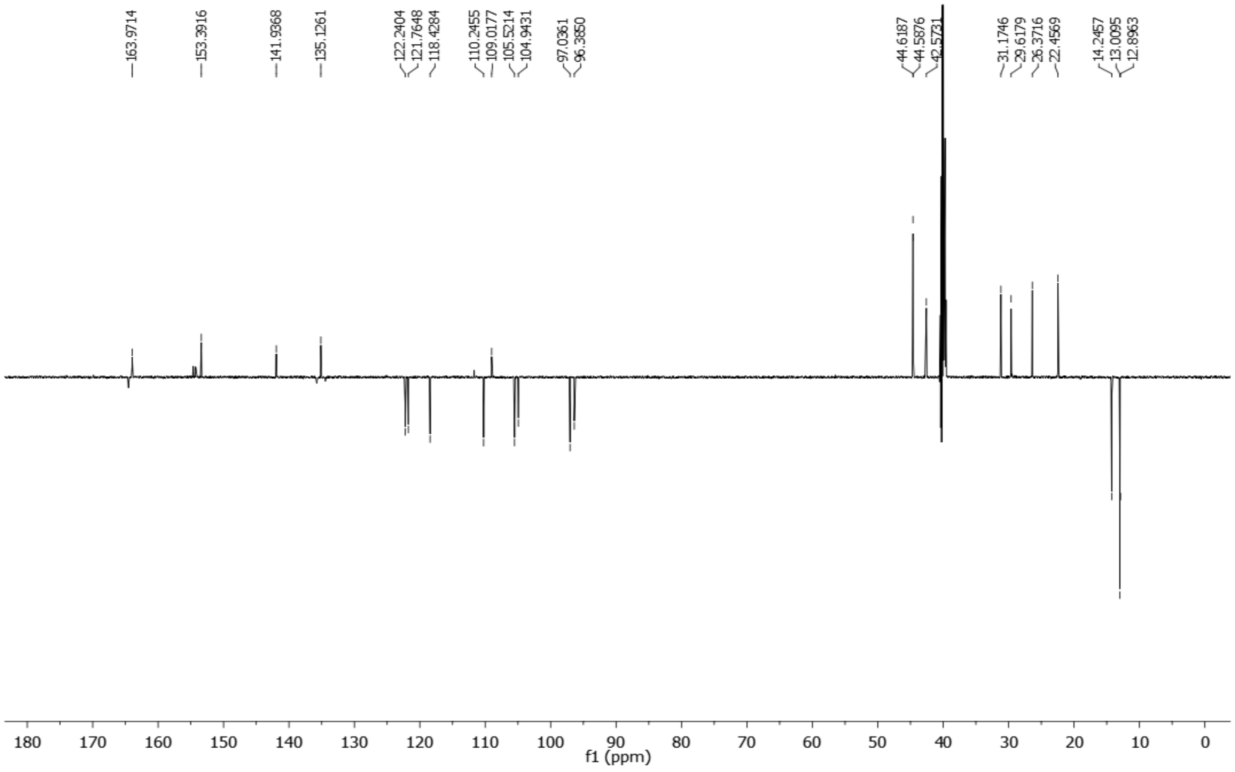

Figure S47. ^{1}H NMR spectrum (DMSO-d_6, 600 MHz) of ***(E)-2-((4-(diethylamino)-2-hydroxybenzylidene)amino)-1-hexyl-1H-benzo[d]imidazole-6-carbonitrile*** ***45***

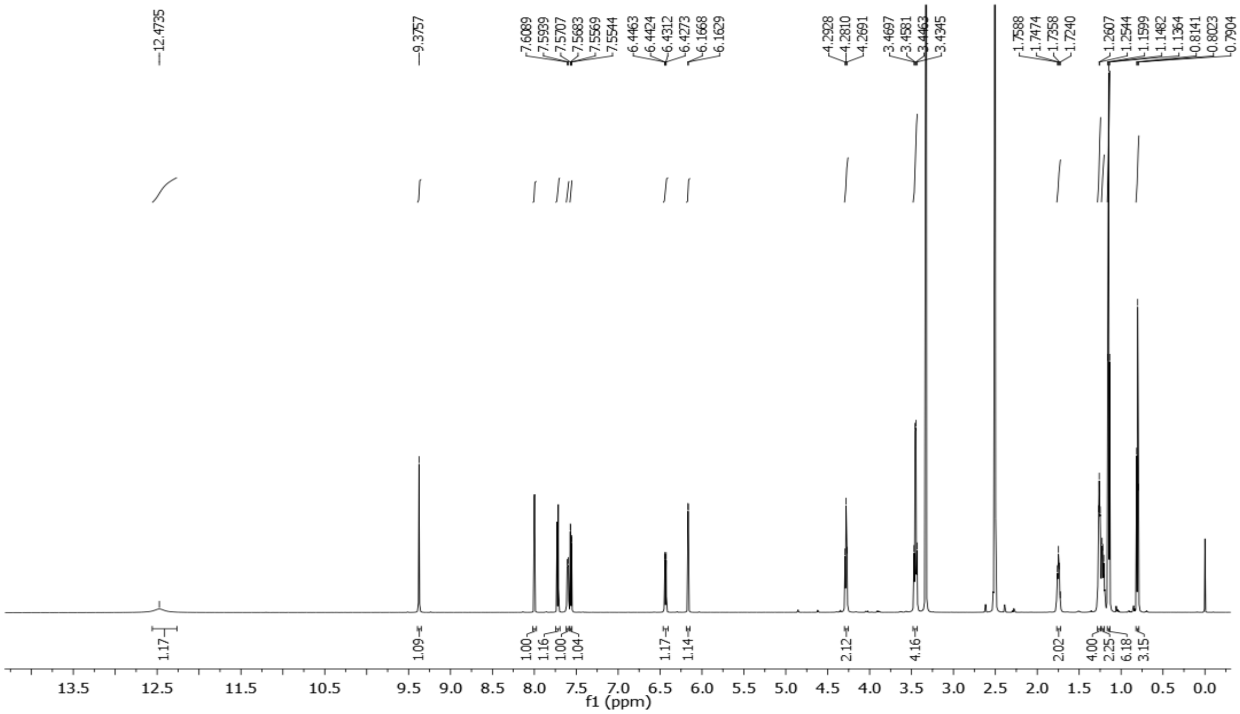

Figure S48. ^{13}C NMR spectrum (DMSO-d_6, 151 MHz) of ***(E)-2-((4-(diethylamino)-2-hydroxybenzylidene)amino)-1-hexyl-1H-benzo[d]imidazole-6-carbonitrile*** ***45***

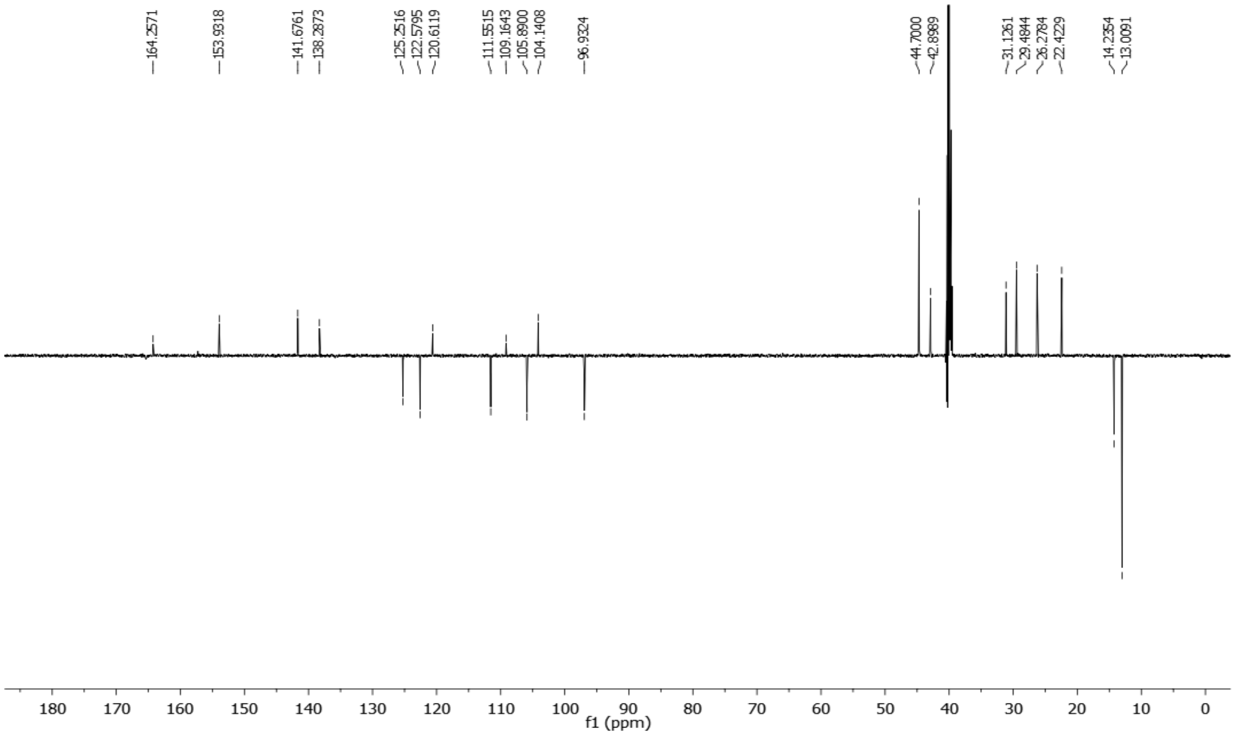

[i] Grgičević, I.; Mikulandra, I.; Bukvić, M.; Banjanac, M.; Radovanović, V.; Habinovec, I.; Bertoša, B.; Novak, P. Discovery of macrozones, new antimicrobial thiosemicarbazone-based azithromycin conjugates: design, synthesis and in vitro biological evaluation. *Int.J. Antimicrob.l Agents* **2020**, *56,* 106147, 5.
